# Dual Energy X-Ray Absorptiometry Body Composition Reference Values from NHANES

**DOI:** 10.1371/journal.pone.0007038

**Published:** 2009-09-15

**Authors:** Thomas L. Kelly, Kevin E. Wilson, Steven B. Heymsfield

**Affiliations:** 1 Hologic, Inc., Bedford, Massachusetts, United States of America; 2 Merck & Co., Rahway, New Jersey, United States of America; Mayo Clinic College of Medicine, United States of America

## Abstract

In 2008 the National Center for Health Statistics released a dual energy x-ray absorptiometry (DXA) whole body dataset from the NHANES population-based sample acquired with modern fan beam scanners in 15 counties across the United States from 1999 through 2004. The NHANES dataset was partitioned by gender and ethnicity and DXA whole body measures of %fat, fat mass/height^2^, lean mass/height^2^, appendicular lean mass/height^2^, %fat trunk/%fat legs ratio, trunk/limb fat mass ratio of fat, bone mineral content (BMC) and bone mineral density (BMD) were analyzed to provide reference values for subjects 8 to 85 years old. DXA reference values for adults were normalized to age; reference values for children included total and sub-total whole body results and were normalized to age, height, or lean mass. We developed an obesity classification scheme by using estabbody mass index (BMI) classification thresholds and prevalences in young adults to generate matching classification thresholds for Fat Mass Index (FMI; fat mass/height^2^). These reference values should be helpful in the evaluation of a variety of adult and childhood abnormalities involving fat, lean, and bone, for establishing entry criteria into clinical trials, and for other medical, research, and epidemiological uses.

## Introduction

The National Health and Nutrition Examination Survey (NHANES) is a program designed to assess the health and nutritional status of adults and children in the United States. NHANES performs a continuous, nationally representative health survey of the civilian, non-institutionalized United States population, collecting data on about 5000 persons each year from interviews, physical examinations, and medical tests including bone densitometry. Previous NHANES surveys provided a widely accepted DXA proximal femur BMD database [1]. In 1999 NHANES began performing DXA whole body measurements on survey subjects 8 years old and older in three mobile examination centers. DXA whole body data from the mobile exam centers was compiled by the NHANES study group and released on the Center for Disease Control (CDC) website. Previous studies used the data to investigate age, gender, and ethnic differences in whole body and regional BMD [2] or compared percent body fat to BMI, waist circumference, and waist-to-stature ratio in adults [3]. We report here on an age, gender, and ethnicity-specific DXA body composition and bone mineral reference database developed from the NHANES survey data collected from 1999 to 2004.

Additionally, we developed an obesity classification scheme by calculating the prevalences of well established BMI classification thresholds and generating similar thresholds for Fat Mass Index (FMI; fat mass/height^2^) [4]. These prevalence-matched FMI classifications should offer superior specificity because the index is based on fat mass, not body weight, which is composed of both fat and lean constituents. The reference data reported here should be helpful in detecting abnormalities in whole body bone and body composition, for establishing reference ranges, for epidemiological considerations (e.g., to establish the prevalence of obesity or sarcopenia), and for entry criteria into clinical trials.

## Methods

### Subjects

Reference curves were developed for the following three major U.S. ethnic groups: Non-Hispanic Whites (hereafter referred to as White), Non-Hispanic Blacks (hereafter referred to as Black), and Mexican Americans. There were not enough observations to develop reference data for other ethnic minorities. Blacks, Mexican Americans, low-income Whites, adolescents between 12 and 19 years old, and subjects 60 years old and older were oversampled to provide more reliable estimates for these groups [3]. Females were excluded from the DXA examination if a pregnancy test was positive at exam time or if they said they were pregnant. Subjects were also excluded if their reported weight exceeded the DXA scan table weight limit of 136 kg or if their reported height was greater than the DXA scan table length of 196 cm”.

### DXA Measurements

The whole body DXA exams in NHANES were acquired according to the procedures recommended by the manufacturer on a QDR 4500A fan beam densitometer (Hologic, Inc., Bedford, MA). All subjects changed into paper gowns and were asked to remove all jewelry and other personal effects that could interfere with the DXA exam. The DXA exams were reviewed and analyzed by the University of California, San Francisco Department of Radiology Bone Density Group using industry standard techniques. Analysis of all exams was performed using Hologic Discovery software version 12.1 in its default configuration. Exams that contained artifacts which could affect the accuracy of the DXA results, such as prosthetic devices, implants or other extraneous objects had the regional and global DXA results for these exams set to missing in the dataset. The precision of the DXA instrument used in the NHANES study has been reported on elsewhere [5], [6], [7].

Body composition measurements are technology and calibration dependent and hence results provided by different instruments vary widely. The DXA instruments used in the NHANES survey employed the calibration proposed by Schoeller et al. [8], whereby DXA lean mass results were calibrated to lean mass measured in 7 independent studies utilizing total body water (4 studies), hydrodensitometry (1 study), and four compartment measures (2 studies). The seven independent studies involved a total of 1195 subjects (602 male, 593 female). The BMD and BMC results were calibrated by the DXA manufacturer and maintained by an internal reference system that periodically measures bone and soft tissue equivalent reference standards during the patient measurement.

The NHANES data sets contained whole body DXA measurements of bone mineral content (BMC, g), areal bone mineral density (BMD, g/cm^2^), fat mass (g) and lean mass including BMC (g) and percent fat, calculated as (fat mass divided by total mass) ×100 along with demographic information for each subject. The above measurements were also available for a number of pre-defined anatomical regions, including the head, arms, legs, trunk, pelvic regions, sub-total whole body (excluding only the head) and whole body. From these whole body measures the following derivative values were calculated: FMI (fat mass/height^2^), lean mass/height^2^, appendicular lean mass/height^2^. For adults, only total body reference values and the above derivative reference values were generated. For children, (subjects less than 20 years of age), total body and sub-total body reference values and selected derivative reference values were generated.

There is increasing realization that fat distribution may be as important as total fat mass, so two indices of fat mass distribution, %fat of the trunk divided by %fat of the legs and fat mass of the trunk divided by fat mass of the limbs (fat mass of arms plus legs) were included in this analysis for adults. These indices may have a role in defining metabolic syndrome or lipodystorphy [9], [10].

### Statistical Methods

Our analysis used the DXA data sets released by NHANES on the Center for Disease Control website http://www.cdc.gov/nchs/about/major/nhanes/dxx/dxa.htm). To prevent bias in the survey due to the fact that the missing data was not completely random, missing data was multiply imputed at the National Center for Health Statistics as described in the technical documentation available on the above referenced website.

The data was partitioned into subgroups according to gender and ethnicity. Ethnicity was self-reported and adjudicated by NHANES into the three major ethnicity groups reported on here (White, Black, and Mexican American). To reduce the complexity of the reference curve fitting procedure, we further divided the data into adult (ages 20–85) and pediatric (ages 8–19+) groups. The number of observations in each subgroup is provided in **Table 1**.

**Table 1:** Number of observations in the reference database by age, gender, and ethnicity.

Age Group	Gender	Whites	Blacks	Mexican Americans
8 to 9	Male	81	90	93
	Female	49	75	51
10 to 11	Male	140	196	169
	Female	97	123	110
12 to 13	Male	186	229	250
	Female	144	167	141
14 to 15	Male	222	292	296
	Female	173	213	213
16 to 17	Male	238	296	308
	Female	154	172	171
18 to 20	Male	338	422	452
	Female	319	333	395
20 to 25	Male	235	138	160
	Female	323	160	239
25 to 30	Male	238	100	164
	Female	338	127	180
30 to 35	Male	241	118	138
	Female	350	145	149
35 to 40	Male	249	114	116
	Female	298	139	135
40 to 45	Male	292	149	164
	Female	260	154	174
45 to 50	Male	244	125	135
	Female	244	148	129
50 to 55	Male	298	101	72
	Female	287	94	100
55 to 60	Male	207	72	63
	Female	204	78	53
60 to 65	Male	248	115	166
	Female	263	138	168
65 to 70	Male	243	112	123
	Female	238	93	144
70 to 75	Male	288	70	105
	Female	236	72	105
75 to 80	Male	225	54	64
	Female	206	65	54
80 to 85	Male	257	23	33
	Female	299	28	32
85+	Male	168	18	17
	Female	184	25	25
Total	Male	4638	2834	3088
	Female	4666	2559	2768

For adult subjects, DXA measures were modeled by gender and ethnicity using age as the independent variable. Whole body fat and lean mass measurements and appendicular lean mass were normalized to height^2^ as suggested by Heymsfield et al. [11].

For pediatric subjects the DXA measures were modeled against age, height, or lean mass as the independent variable. The development of skeletal reference values were based on the recommendations of the ICSD task force for Pediatric Official Positions paper [12]. Additional soft tissue reference values generated in pediatric subjects included total body %fat and lean mass/height^2^ (kg/m^2^).

A curve fitting procedure called LMS (lmsChartMaker Pro Version 2.3) [13] was used to generate the reference curves because it is capable of handling the relatively common situation where the underlying reference data are skew, i.e. the data are not normally distributed. It does so by normalizing the underlying reference data by dividing the independent measure (e.g. age) into groups and then applying a power transformation which extends one tail of the distribution and contracts the other, eliminating skewness in the variable under analysis. A smooth curve is fitted to the normalizing power transformation for each age group, generating an optimum “L” (power) curve that normalizes the dependent measure, e.g. %fat, over the entire age range. The procedure also fits Median (M) and coefficient of variation (S) curves, and these three curves (L, M, and S) fully describe the reference data. We report the more commonly used population standard deviation, σ, which is S times M. The z-scores can be calculated by the following equation:
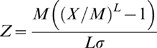
or
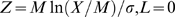
where X is the physical measurement (e.g. DXA Total Body BMD, DXA %fat, etc.), L is the power transformation, M is the median value, and σ is the population standard deviation [13]. Percentiles can be obtained from z-scores, e.g. z-scores of −1.881, −1.645, −0.674, 0, 0.674, 1.645, and 1.881 correspond to the 3rd, 5th, 25th, 50th, 75th, 95th, and 97th percentiles, respectively.

As previously stated, the data were separated into pediatric and adult groups to simplify the curve fitting process and to lower the equivalent degrees of freedom required to fit a suitable curve to the reference data. For example, it was observed that very complex curves were required to produce a satisfactory model of BMC versus age in the entire population, due to the exponential BMC accrual observed in younger subjects combined with a consolidation phase of BMC in early adulthood and then a relatively modest decline in BMC that occurs during aging. When these two very different processes (growth and aging) were modeled independently, much simpler models could be employed with improved goodness of fit and decreased complexity.

During the curve fitting process, the weighted observations from the NHANES data sets were fitted by selecting more parsimonious models over more complex models so long as the goodness of fit was similar, i.e. we tried to avoid over fitting the curves. Further, we employed careful visual inspection of the Q statistic, a plot of standardized residuals in which the data are split into groups and the non-random between group variations in the estimated moments of the z-scores are plotted against the equivalent degrees of freedom used to fit the curve. The Q-statistic was considered satisfactory at or below a value of 2 for the L, M, and S curves and if the fitted curve was reasonably smooth and plausible for the data being fitted, as recommended by the developers of LMS [13].

An obesity classification scheme based on FMI was developed by first calculating the prevalences of established WHO BMI classification thresholds (e.g. normal, overweight, obese) in the present NHANES survey data using “young normal adults” at age of 25. From these prevalence values for BMI, we then generated classification thresholds for FMI that gave the same prevalences as BMI in this population at age 25. In effect we have translated the WHO BMI thresholds into FMI equivalent values by matching the prevalences of the two indices at each classification cut-off point. The FMI classifications should misclassify fewer individuals because FMI is based on fat mass, not body weight, which contains both fat and lean components and therefore misclassifies some muscular subjects as overweight or obese.

## Results

The number of observations in the reference database by age group, gender, and ethnicity is provided in Table 1. The reference curves developed from the whole body DXA measures and derivative values from the 2008 NHANES data set are provided in Table 2. Reference values for each of the DXA measures in Table 2 are provided in supplementary Table S1 through Table S20 by sex and ethnicity. Scatter plots with the fits including the mean, the 3rd and 97^th^ percentiles superimposed upon the raw data values are provided in the supplementary Figure S1 through Figure S20 for adults and children.

**Table 2:** List of reference curves generated from the 2008 NHANES DXA whole body data set.

DXA Measure	Independent Variable	Age Group	Supplemental Table and Figure
Fat Mass/Height^2^ (FMI)	Age	Adult Only	S1
Total Body % Fat	Age	Adult and Pediatric	S2 and S9
% Fat Trunk/% Fat Legs	Age	Adult Only	S3
Trunk/Limb Fat Mass Ratio	Age	Adult Only	S4
Lean Mass/Height^2^	Age	Adult and Pediatric	S5 and S10
Appendicular Lean Mass/Height^2^	Age	Adult Only	S6
Total Body BMD	Age	Adult and Pediatric	S7 and S11
Total Body BMC	Age	Adult and Pediatric	S8 and S12
Sub-total Body BMD (excludes head)	Age	Pediatric Only	S13
Sub-total Body BMC (excludes head)	Age	Pediatric Only	S14
Total Body BMD	Height	Pediatric Only	S15
Total Body BMC	Height	Pediatric Only	S16
Sub-total Body BMD (excludes head)	Height	Pediatric Only	S17
Sub-total Body BMC (excludes head)	Height	Pediatric Only	S18
Total Lean Mass	Height	Pediatric Only	S19
Sub-total Body BMC (excludes head)	Total Lean Mass	Pediatric Only	S20

For each whole body DXA measure in column 1, male and female reference curves for White, Black, and Mexican American subjects were modeled against the independent variable in column 2. Adult age range is 20 to 85 years; Pediatric age range is 8 to 20 years.

As expected, significant differences were observed between genders for the various measures and these differences varied with age. The median %fat increased monotonically with age from 17 to 85 in males, while in women it peaked at approximately age 65. Differences between ethnicities were more modest, and varied with gender. Black males had slightly less %fat than non-black males at all ages. Differences in %fat between non-black males and non-black females was about 11–12% and fairly constant with age; larger differences of 12–16% were observed between black males and black females and the differences were more variable with age.

As can be seen from the scatter plots in the Supplementary Figures, some of the data were not only significantly skewed, but the degree of skewness and the standard deviation varied with the independent variable. The curve fitting method of LMS smoothly models both varying non-normal distributions and varying standard deviations to construct reference curves which accurately model the true distribution and variance of the underlying data. The LMS curve fitting procedure adjusts for skewness so that the percentile values and z-scores generated by the LMS values are robust when the data are not normally distributed. Statistical theory states that a properly fitted reference curve will generate z-scores very close to zero with a standard deviation very close to unity. Using the SAS system, we calculated z-scores for all subjects and all DXA measures in the NHANES dataset; average z-scores were very close to zero with standard deviations very close to unity for all fitted DXA measures, indicating that the LMS curve fitting procedure produced robust, unbiased fits to the underlying reference data.

We employed the BMI classifications from the WHO technical report on the use and interpretation of anthropometry [14], [15] to establish similar classifications for FMI by calculating the prevalences at age 25 for each of the WHO BMI classification thresholds (e.g. mild thinness, normal, overweight, obese class 1, etc.) and then assigning FMI values with matching prevalences to each classification threshold. The FMI classification ranges were similar for the three ethnic groups even though the BMI classification prevalences varied widely (see Table 3). However, large differences in FMI thresholds were observed between genders, with women having higher FMI for all classification categories, requiring the use of gender-specific FMI thresholds. The final FMI classification thresholds in Table 4 were based on the values for White subjects in Table 3. Although the values for the three ethnic groups were similar, there were many more observations for White subjects and hence the use of these values should provide more robust classification estimates.

**Table 3:** FMI (kg/m^2^) thresholds with the same prevalence as a given BMI threshold at age 25.

Sex	Ethnicity	FMI matching BMI<16 (prevalence)	FMI matching BMI<17 (prevalence)	FMI matching BMI<18.5 (prevalence)	FMI matching BMI>25 (prevalence)	FMI matching BMI>30 (prevalence)	FMI matching BMI>35 (prevalence)	FMI matching BMI>40 (prevalence)
M	White	<1.9 (0.1%)	<2.3 (0.5%)	<2.9 (2.6%)	>6.0 (55%)	>8.9 (22%)	>11.9 (8%)	>15.0 (2.6%)
M	Black	<1.7 (0.2%)	<2.0 (0.7%)	<2.5 (3.3%)	>5.4 (54%)	>8.1 (24%)	>11.2 (11%)	>14.4 (3.3%)
M	Mexican American	<2.0 (<0.1%)	<2.3 (0.1%)	<3.0 (0.6%)	>6.3 (59%)	>9.2 (20%)	>12.3 (6%)	>15.4 (1.7%)
F	White	<3.5 (0.8%)	<4.0 (2.2%)	<4.9 (7%)	>9.2 (47%)	>12.9 (21%)	>16.8 (9%)	>20.6 (4.1%)
F	Black	<3.4 (0.5%)	<3.9 (1.1%)	<4.7 (3.0%)	>8.6 (70%)	>11.9 (42%)	>15.3 (22%)	>18.7 (11%)
F	Mexican American	<3.8 (0.1%)	<4.3 (0.5%)	<5.2 (2.2%)	>9.4 (62%)	>12.8 (29%)	>16.1 (12%)	>19.2 (4.6%)

The above FMI thresholds gave the same prevalence as BMI for each of the principal BMI classification cut-off points. FMI thresholds were similar among the ethnic groups even though their prevalences vary considerably.

**Table 4:** Fat Mass Index (kg/m^2^) classification ranges.

FMI Class	Severe Fat Deficit	Moderate Fat Deficit	Mild Fat Deficit	Normal	Excess Fat	Obese Class I	Obese Class II	Obese Class III
M	<2	2 to <2.3	2.3 to <3	3–6	>6 to 9	>9 to 12	>12 to 15	>15
F	<3.5	3.5 to <4	4 to <5	5–9	>9 to 13	>13 to 17	>17 to 21	>21

Classification ranges for FMI that match the prevalences of the WHO BMI classifications (see Table 3). Unlike BMI, FMI is a gender specific measure of fat not confounded by lean tissue.

## Discussion

The lack of representative body composition reference values has limited their potential application in clinical and research settings. The current report provides the first reference values on a nationally representative NHANES data set acquired using well established DXA technology. These reference values should prove useful for many applications previously limited by inadequate or unavailable reference data. The NHANES reference values reported here are only directly compatible with Hologic fan beam DXA scanners operating software version 12.5 or higher utilizing the NHANES calibration. The NHANES reference data used in this study included multiply imputed data and this may be a limitation of the study. As with any reference database, the application of these data to generate diagnostic scores for a given patient or patient population should be performed with appropriate measure of forethought and caution.

The reference data presented here may be useful in detecting skeletal and body composition abnormalities in children arising from a wide variety of conditions and chronic diseases including anorexia nervosa, growth hormone deficiency, glucocorticoid use, immobilization, cystic fibrosis, hypogonadism, thalassemia, malnutrition, weight management, chronic inflammatory diseases, endocrine disturbances, childhood cancer, transplantation, and other disorders [16]. Increasing numbers of children are being referred for DXA whole body measurements because of its ability to evaluate global and regional bone mineral and body composition. Total body less head (sub-total) whole body results are currently recommended by the ISCD for the evaluation of some childhood disorders [12]. Normalizing to height is appropriate in children with delayed growth and maturation. Furthermore, sub-total (whole body less head) BMD and BMC reference data are necessary because the head is disproportionately large in young children and may mask deficits at other skeletal sites. As a result, we developed whole body and sub-total BMD and BMC reference curves for subjects 8 to 20 years old normalized to both age and height. Height is an important body size adjustment in children because children mature at different rates and because many chronically ill children are small for their age. As a result, chronological age may not be the best indicator of a child's growth and development and therefore age-matched comparisons may not be appropriate. The International Society for Clinical Densitometry (ISCD) also recommends the use of a sufficiently large sample of the general population that takes into consideration gender, age, and ethnicity [17]. The present database meets all of these conditions through the use of gender, and ethnicity specific reference data from NHANES including DXA sub-total body measures normalized to age and to body size (height). We also developed total lean mass versus height and sub-total BMC versus lean mass reference curves for children, as suggested by the ISCD in its latest Official Positions Statement for Pediatric DXA (see www.iscd.org). These two additional curves should allow for the detection of abnormalities in lean mass accrual, e.g. in growth hormone deficient children, and for the detection of abnormalities in the bone-muscle unit, respectively, as suggested by Schoenau et al. [18]. NHANES did not collect information on pubertal development and therefore no adjustment to the reference values to account for delayed maturation was possible. As a result, clinicians should use caution when interpreting DXA measures in subjects with delayed or advanced maturation.

In adults, we normalized both fat and lean mass by height^2^, just like BMI, which is simply weight divided by height^2^. Studies have shown that lean mass and weight scale with height to approximately the power of two, establishing an analytic framework for height-scaled indices [11]. The same study also found that fat mass scaled to height^2^, although the association was weaker. The present study confirmed this observation. We hypothesized that comparison of a subject's FMI value to healthy young normal FMI values may be useful in the diagnosis and management of clinical obesity, for identifying subjects with high obesity-disease risks, and for enrolling high risk subjects in clinical trials. However, while these cross sectional data enable this definition, whether this definition is useful and appropriate will have to be investigated in future studies which look at obesity related morbidity and outcomes. Defining thresholds in comparison to a young adult population has been adopted in the field of osteoporosis research (e.g. the bone mineral density T-score) and has proven itself a useful tool.

Reference curves for appendicular lean mass divided by height^2^ were developed because this DXA measure is a good surrogate for skeletal muscle mass and a possible index of sarcopenia [19]. DXA is the only widely available technology capable of providing regional measures of fat and lean mass, and it has been shown that fat and lean distribution may predict health outcomes. A study of elderly subjects [13] demonstrated that sarcopenia, defined as appendicular skeletal muscle mass (kg/height^2^) less than two standard deviations below the mean of a young reference group, predicted self-reported physical disability in elderly men and women independent of other covariates such as age, obesity, ethnicity, and income level.

In the present NHANES database fat comprises approximately 24% of body weight in males and 38% in females at age 25. Although these %fat values may seem quite high, they are consistent with the %fat reference ranges reported by NHANES in the 1988–1994 survey that utilized bioimpedence analysis. In the 1988–1994 survey, the average male and female %fat values at age 25 were 23% and 34%, respectively [20]. The slightly higher %fat values reported here are consistent with the secular trend of increasing weight and BMI in adult Americans. However, the two studies used different technologies to measure %fat, so direct comparisons between the two studies are difficult to interpret.

DXA is capable of separating body mass into fat and lean components, thereby permitting the evaluation of fat mass without the confounding influence of other tissue constituents. We propose the use of FMI (fat mass/height^2^) as a measure of abnormally low or excess fat mass because FMI evaluates only the fat mass component of body weight. Using FMI, abnormalities in fat mass can be assessed without interference from other unrelated components such as excesses or deficits of muscle or water.

Our data reveal %fat and FMI increasing up until about age 80 in men and age 65 in women. Increasing adiposity is an unhealthy trend and hence comparing DXA measurements of %fat and FMI to age-matched peers may not be the most appropriate approach. We postulate that it may be better to compare adults to young normal gender and ethnicity-matched subjects at age 25. For example, using the median value of %fat for non-Hispanic white males at age 25 as an arbitrary “healthy target”, only 25% of 45 year old non-Hispanic white males are at or below this target, and by age 69, the number falls to less than 10%. Though alarming, the low percentage of subjects at or below this healthy %fat target probably provides a more realistic assessment of %fat and FMI levels versus comparing subjects to age-matched controls, where by definition 50% of subjects would appear “normal”.

We selected from the present data a young adult group with a BMI between 18.5 and 25 to establish reasonably robust reference values for “normal” FMI. Following this same methodology, we developed classifications for FMI by selecting values for FMI that matched the population prevalence of the WHO BMI classifications [14] in young adults at age 25. For example, at age 25 a FMI value of greater than 6 kg/m^2^ for men and 9 kg/m^2^ for women matches the same prevalence value of “overweight” obtained with a BMI of 25, the BMI cut-off point for “overweight”. Similarly, FMI values of greater than or equal to 9 kg/m^2^ for men and 13 kg/m^2^ for women defines the same obesity prevalence as a BMI of 30 in this population. Kyle et al. [21] employed similar methodology to generate FMI classifications corresponding to low, normal, overweight, and obese BMI categories. Note that although the same prevalences were used for each classification, FMI and BMI actually classify different subjects into the various categories. Were this not so, it would not be possible for one method to have an advantage over the other. Gallagher et al. [22] used the same approach to generate guidelines for healthy percent fat ranges based on BMI.

A major shortcoming of BMI is that it provides a measure of excess weight, not excess fat. Another obvious limitation of BMI is that it does not account for gender or ethnicity. Table 3 clearly demonstrates that BMI prevalences are heavily influenced by both gender and ethnicity. At age 25, the FMI data in Table 3 indicates that there are substantial differences in adiposity between genders, with mean values for women ranging from 8.9 to 10.9 kg/m^2^ and mean values for males between 5.6 to 6.8 kg/m^2^ for the three ethnic groups. From these data it appears likely that lacking gender or ethnicity adjustments, BMI may be overestimating obesity in some groups and underestimating it in others. Furthermore, subjects with a high degree of muscularity, e.g. body builders, are often misclassified as “overweight” or “obese” by BMI; these same subjects would probably not fall into an abnormal classification range with FMI because their excess weight is mostly lean mass. Percent body fat (%fat) measurements are also complicated by increased muscularity, but here the bias is in the opposite direction, as increases in muscle mass offset increases in fat mass, making a %fat measurement appear more or less normal.

We suggest that using these proposed FMI values for overweight and obese classifications will result in fewer misclassifications than either BMI or %fat. The FMI classifications in Table 4 should be considered guidelines that may misclassify fewer individuals than BMI because they are based on fat mass instead of weight. It is also worth noting that other technologies which measure fat mass will have to be calibrated to the DXA systems used in this study in order to make uses of these FMI classifications.

Whether or not the use of the proposed FMI classification scheme will confer benefits over BMI in terms of predicting obesity-related morbidity or mortality will have to be investigated in future studies. The FMI classifications presented here are based on prevalence data, not disease risk, and therefore the clinical utility of the FMI classification scheme will not be known until data relating disease risk to FMI becomes available.

## Supporting Information

Figure S1 Fat Mass/Height^2^ (kg/m^2^) vs. Age in adults.
(0.34 MB PDF)

Figure S2 Percent Fat (%) vs. Age in adults.
(0.31 MB PDF)

Figure S3 % Fat Trunk/%Fat Legs vs. Age in adults.
(0.34 MB PDF)

Figure S4 Trunk to Limb Fat Mass Ratio vs. Age in adults.
(0.55 MB PDF)

Figure S5 Lean Mass/Height^2^ (kg/m^2^) vs. Age in adults.
(0.34 MB PDF)

Figure S6 Appendicular Lean Mass/Height^2^ (kg/m^2^) vs. Age in adults.
(0.30 MB PDF)

Figure S7 Total Body BMD (g/cm^2^) vs. Age in adults.
(0.31 MB PDF)

Figure S8 Total Body BMC (g) vs. Age in adults.
(0.57 MB PDF)

Figure S9 Percent Fat (%) vs. Age in pediatrics.
(0.18 MB PDF)

Figure S10 Lean Mass/Height^2^ (kg/m^2^) vs. Age in adults.
(0.20 MB PDF)

Figure S11 Total Body BMD (g/cm^2^) vs. Age in Pediatrics.
(0.22 MB PDF)

Figure S12 Total Body BMC (g) vs. Age in pediatrics.
(0.23 MB PDF)

Figure S13 Sub-total Body BMD (g/cm^2^) vs. Age in Pediatrics.
(0.51 MB PDF)

Figure S14 Sub-total Body BMC (g) vs. Age in pediatrics.
(0.23 MB PDF)

Figure S15 Total Body BMD (g/cm^2^) vs. Height (cm) in pediatrics.
(0.51 MB PDF)

Figure S16 Total Body BMC (g) vs. Height (cm) in pediatrics.
(0.24 MB PDF)

Figure S17 Sub-total Body BMD (g/cm^2^) vs. Height (cm) in pediatrics.
(0.24 MB PDF)

Figure S18 Sub-total BMC (g) vs. Height (cm) in pediatrics.
(0.25 MB PDF)

Figure S19 Total Lean Mass (g) vs. Height (cm) in pediatrics.
(0.25 MB PDF)

Figure S20 Sub-total BMC (g) vs. Total Lean Mass (g) in pediatrics.
(0.27 MB PDF)

Table S1 Fat Mass/Height^2^ (kg/m^2^) vs. Age in adult subjects.
(0.08 MB DOC)

Table S2 %Fat (%) vs. Age in adult subjects.
(0.08 MB DOC)

Table S3 %Fat Trunk/%Fat Legs vs. Age in adult subjects.
(0.08 MB DOC)

Table S4 Trunk to Limb Fat Mass Ratio vs. Age in Adult subjects.
(0.08 MB DOC)

Table S5 Lean Mass/Height^2^ (kg/m^2^) vs. Age in adult subjects.
(0.08 MB DOC)

Table S6 Appendicular Lean Mass/Height^2^ (kg/m^2^) vs. Age in adult subjects.
(0.08 MB DOC)

Table S7 Total Body BMD (g/cm^2^) vs. Age in adult subjects.
(0.08 MB DOC)

Table S8 Total Body BMC (g) vs. Age in adult subjects.
(0.08 MB DOC)

Table S9 %Fat (%) vs. Age in pediatric subjects.
(0.05 MB DOC)

Table S10 Lean Mass/Height^2^ (kg/m^2^) vs. Age in pediatric subjects.
(0.05 MB DOC)

Table S11 Total Body BMD (g/cm^2^) vs. Age in pediatric subjects.
(0.05 MB DOC)

Table S12 Total Body BMC (g) vs. Age in Pediatric subjects.
(0.05 MB DOC)

Table S13 Sub-total Body BMD (g/cm^2^) vs. Age in pediatric subjects.
(0.05 MB DOC)

Table S14 Sub-total Body BMC (g) vs. Age in pediatric subjects.
(0.05 MB DOC)

Table S15 Total Body BMD (g/cm^2^) vs. Height (cm) in pediatric subjects.
(0.08 MB DOC)

Table S16 Total Body BMC (g) vs. Height (cm) in pediatric subjects.
(0.09 MB DOC)

Table S17 Sub-total Body BMD (g/cm^2^) vs. Height (cm) in pediatric subjects.
(0.08 MB DOC)

Table S18 Sub-total BMC (g) vs. Height (cm) in pediatric subjects.
(0.08 MB DOC)

Table S19 Total Lean Mass (g) vs. Height (cm) in pediatric subjects.
(0.08 MB DOC)

Table S20 sub-total BMC (g) vs. Total Lean Mass (g) in pediatric subjects.
(0.09 MB DOC)
