# Supplementary material for: Dual Energy X-Ray Absorptiometry Body Composition Reference Values from NHANES
Source: PLoS One. 2009 Sep 15;4(9):e7038. doi: 10.1371/journal.pone.0007038 (PMC2737140; doi:10.1371/journal.pone.0007038)

Figure S3: % Fat Trunk / % Fat Legs vs. Age in adults. Solid lines indicate the 3rd, 50th, and 97th percentiles.

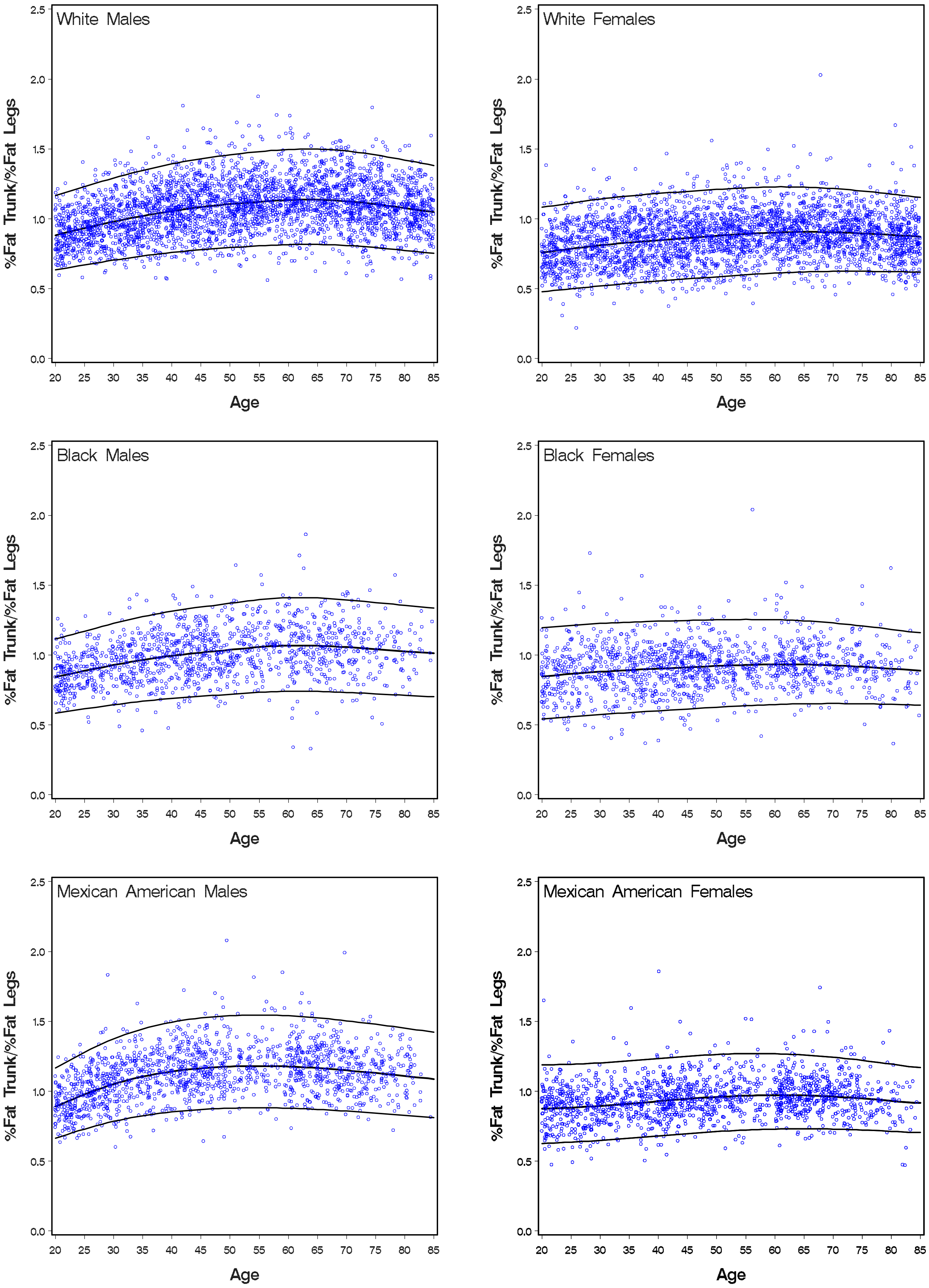

Supplement: Figure S3 — % Fat Trunk/%Fat Legs vs. Age in adults. (0.34 MB PDF) [file pone.0007038.s003.pdf]
