# Supplementary material for: Dual Energy X-Ray Absorptiometry Body Composition Reference Values from NHANES
Source: PLoS One. 2009 Sep 15;4(9):e7038. doi: 10.1371/journal.pone.0007038 (PMC2737140; doi:10.1371/journal.pone.0007038)

Figure S4: Trunk to Limb Fat Mass Ratio vs. Age in adults. Solid lines indicate the 3rd, 50th, and 97th percentiles.

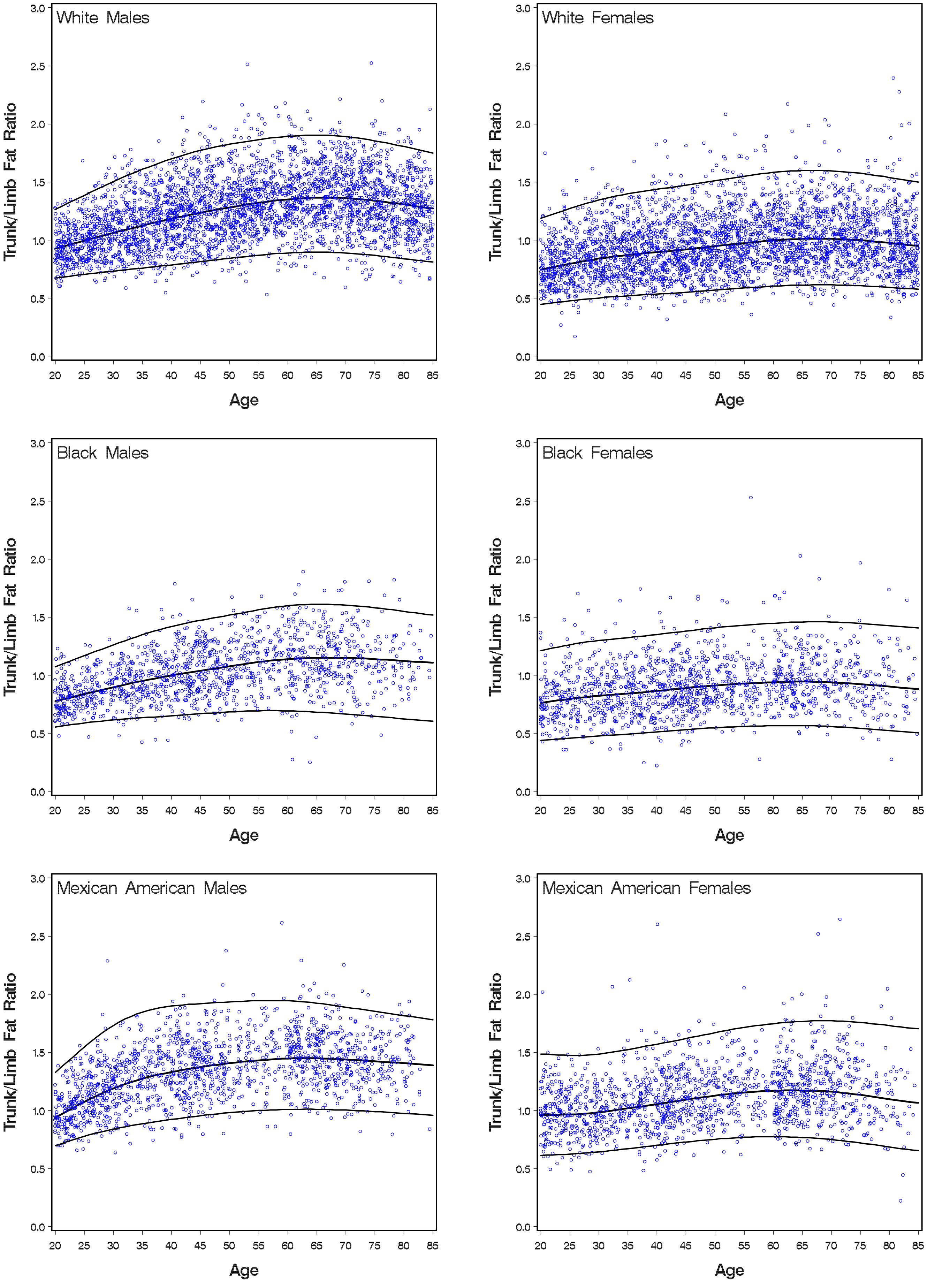

Supplement: Figure S4 — Trunk to Limb Fat Mass Ratio vs. Age in adults. (0.55 MB PDF) [file pone.0007038.s004.pdf]
