# Supplementary material for: Dual Energy X-Ray Absorptiometry Body Composition Reference Values from NHANES
Source: PLoS One. 2009 Sep 15;4(9):e7038. doi: 10.1371/journal.pone.0007038 (PMC2737140; doi:10.1371/journal.pone.0007038)

Figure S9: Percent Fat (%) vs. Age for pediatrics. Solid lines indicate the 3rd, 50th, and 97th percentiles.

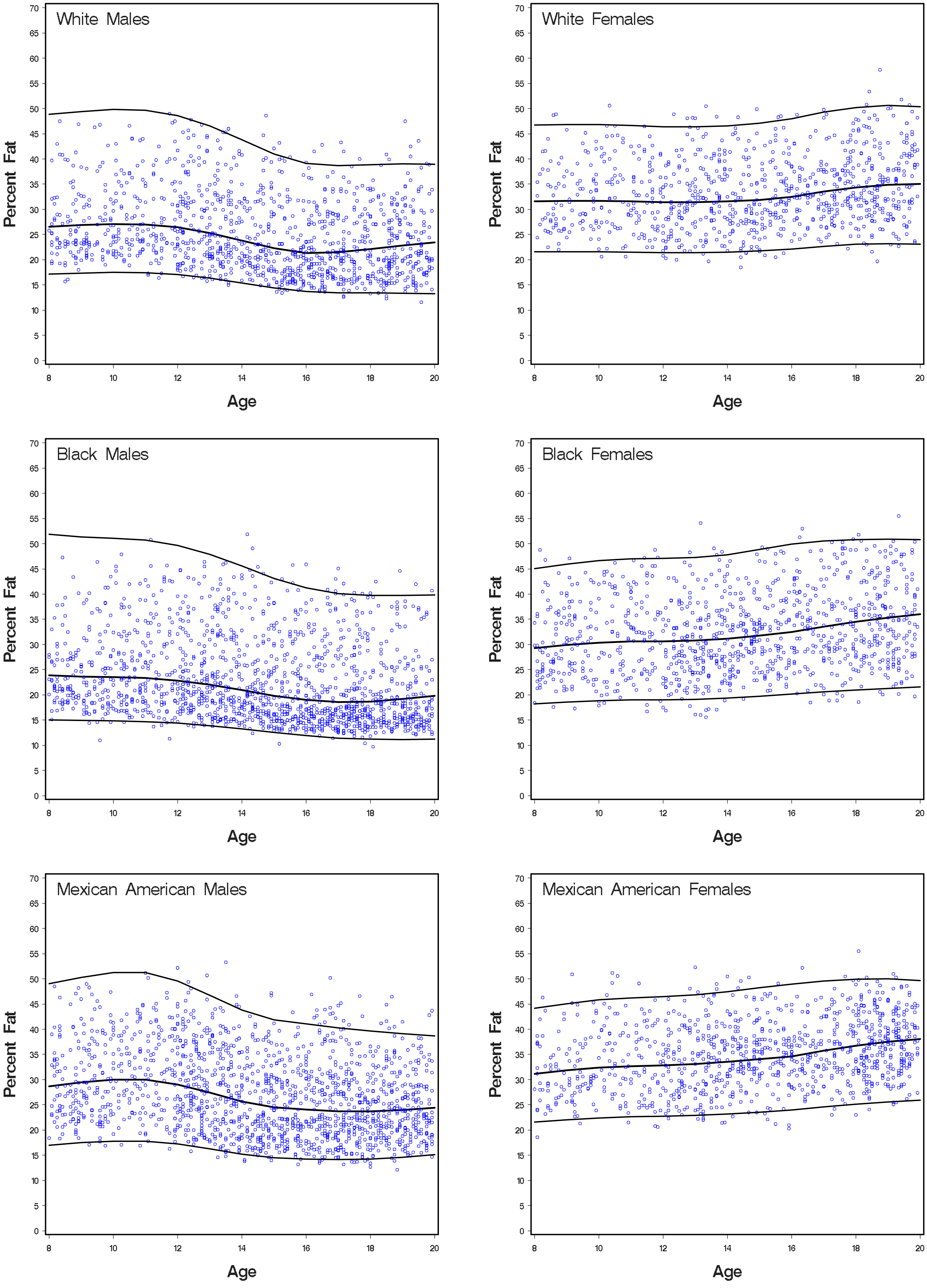

Supplement: Figure S9 — Percent Fat (%) vs. Age in pediatrics. (0.18 MB PDF) [file pone.0007038.s009.pdf]
