# Supplementary material for: Dual Energy X-Ray Absorptiometry Body Composition Reference Values from NHANES
Source: PLoS One. 2009 Sep 15;4(9):e7038. doi: 10.1371/journal.pone.0007038 (PMC2737140; doi:10.1371/journal.pone.0007038)

Figure S20: Sub-total (excludes head) BMC (g) vs. Total Lean Mass (g) in pediatrics. Solid lines indicate the 3rd, 50th, and 97th percentiles.

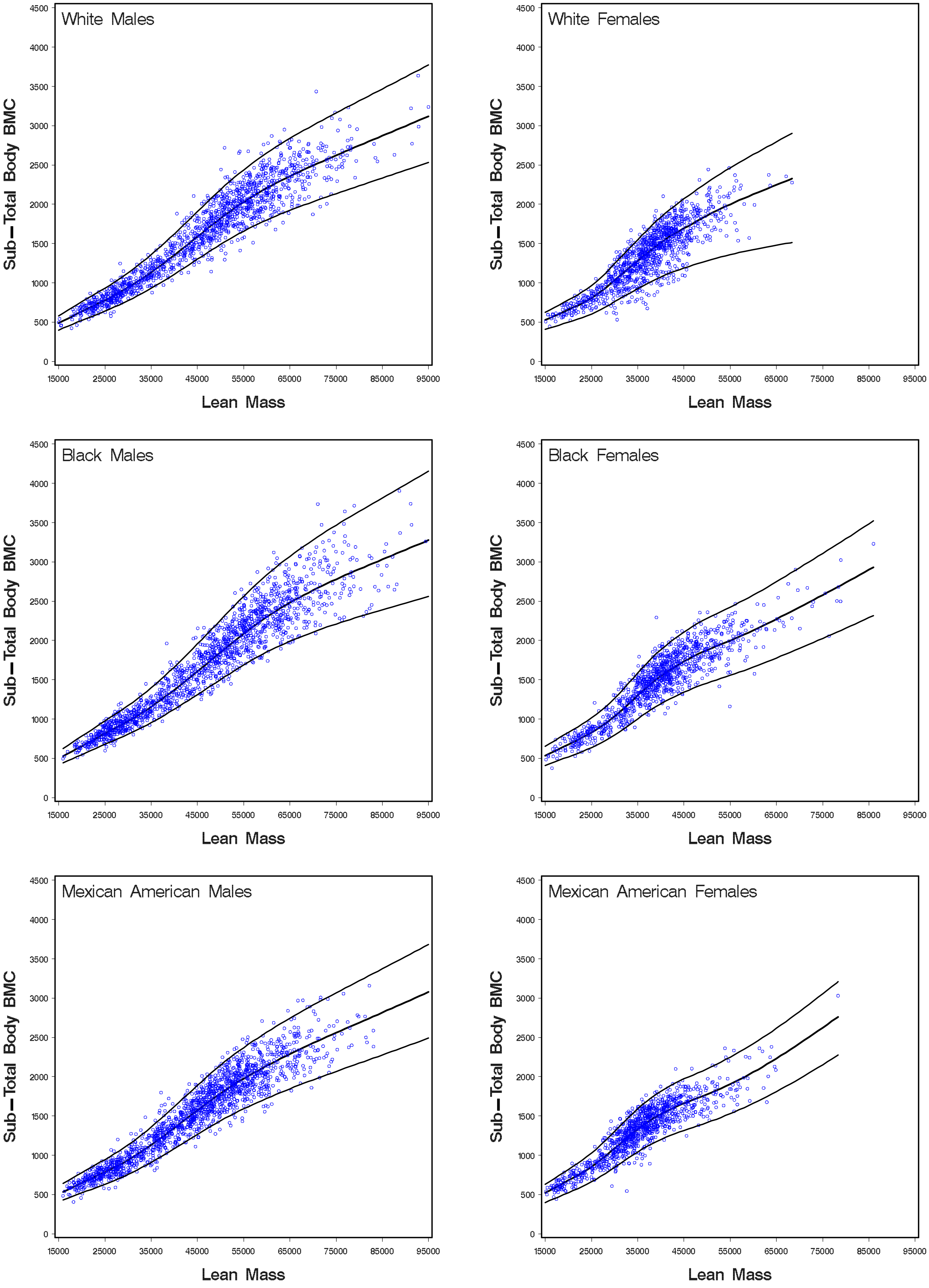

Supplement: Figure S20 — Sub-total BMC (g) vs. Total Lean Mass (g) in pediatrics. (0.27 MB PDF) [file pone.0007038.s020.pdf]
