# Supplementary material for: Dual Energy X-Ray Absorptiometry Body Composition Reference Values from NHANES
Source: PLoS One. 2009 Sep 15;4(9):e7038. doi: 10.1371/journal.pone.0007038 (PMC2737140; doi:10.1371/journal.pone.0007038)
Supplement: Table S1 — Fat Mass/Height2 (kg/m2) vs. Age in adult subjects. (0.08 MB DOC) [file pone.0007038.s021.doc]

Table S1: Fat Mass/Height^2^ (kg/m^2^) vs. Age in adult subjects.

| **Males** | | | | | | | | | | | | | | |
| --- | --- | --- | --- | --- | --- | --- | --- | --- | --- | --- | --- | --- | --- | --- |
|  | | White | | |  | | Black | | |  | | Mexican American | | |
| Age | | M | σ | L |  | | M | σ | L |  | | M | σ | L |
| 20 | | 5.95 | 2.59 | -0.144 |  | | 4.82 | 2.49 | -0.378 |  | | 5.89 | 2.16 | -0.238 |
| 25 | | 6.37 | 2.69 | -0.114 |  | | 5.59 | 2.81 | -0.299 |  | | 6.80 | 2.44 | -0.173 |
| 30 | | 6.78 | 2.77 | -0.084 |  | | 6.17 | 3.02 | -0.220 |  | | 7.45 | 2.60 | -0.108 |
| 35 | | 7.19 | 2.84 | -0.054 |  | | 6.56 | 3.11 | -0.141 |  | | 7.84 | 2.67 | -0.044 |
| 40 | | 7.57 | 2.89 | -0.024 |  | | 6.81 | 3.14 | -0.062 |  | | 8.06 | 2.67 | 0.019 |
| 45 | | 7.92 | 2.92 | 0.006 |  | | 6.98 | 3.12 | 0.017 |  | | 8.19 | 2.64 | 0.080 |
| 50 | | 8.21 | 2.91 | 0.037 |  | | 7.15 | 3.10 | 0.095 |  | | 8.35 | 2.62 | 0.141 |
| 55 | | 8.49 | 2.90 | 0.067 |  | | 7.42 | 3.12 | 0.174 |  | | 8.51 | 2.60 | 0.200 |
| 60 | | 8.71 | 2.86 | 0.097 |  | | 7.72 | 3.14 | 0.252 |  | | 8.64 | 2.57 | 0.260 |
| 65 | | 8.84 | 2.78 | 0.127 |  | | 7.95 | 3.13 | 0.331 |  | | 8.67 | 2.50 | 0.319 |
| 70 | | 8.82 | 2.66 | 0.157 |  | | 8.10 | 3.08 | 0.409 |  | | 8.54 | 2.39 | 0.378 |
| 75 | | 8.68 | 2.50 | 0.187 |  | | 8.16 | 2.99 | 0.488 |  | | 8.30 | 2.26 | 0.437 |
| 80 | | 8.46 | 2.32 | 0.217 |  | | 8.17 | 2.89 | 0.567 |  | | 8.02 | 2.11 | 0.496 |
| 85 | | 8.20 | 2.14 | 0.248 |  | | 8.15 | 2.78 | 0.640 |  | | 7.72 | 1.97 | 0.556 |
| **Females** | | | | | | | | | | | | | | |
|  | White | | | |  | Black | | | |  | Mexican American | | | |
| Age | M | | σ | L |  | M | | σ | L |  | M | | σ | L |
| 20 | 8.48 | | 3.80 | -0.310 |  | 10.02 | | 4.56 | -0.048 |  | 9.89 | | 3.54 | -0.073 |
| 25 | 8.90 | | 3.89 | -0.249 |  | 10.87 | | 4.81 | 0.003 |  | 10.51 | | 3.70 | -0.047 |
| 30 | 9.35 | | 3.98 | -0.188 |  | 11.59 | | 4.98 | 0.054 |  | 11.07 | | 3.82 | -0.021 |
| 35 | 9.82 | | 4.07 | -0.126 |  | 12.09 | | 5.05 | 0.106 |  | 11.52 | | 3.90 | 0.005 |
| 40 | 10.27 | | 4.13 | -0.064 |  | 12.59 | | 5.10 | 0.157 |  | 11.98 | | 3.98 | 0.031 |
| 45 | 10.72 | | 4.19 | -0.003 |  | 13.01 | | 5.11 | 0.210 |  | 12.46 | | 4.06 | 0.057 |
| 50 | 11.20 | | 4.25 | 0.059 |  | 13.33 | | 5.08 | 0.262 |  | 12.80 | | 4.09 | 0.083 |
| 55 | 11.67 | | 4.29 | 0.121 |  | 13.57 | | 5.00 | 0.316 |  | 12.91 | | 4.04 | 0.109 |
| 60 | 12.03 | | 4.28 | 0.183 |  | 13.68 | | 4.88 | 0.369 |  | 12.88 | | 3.95 | 0.135 |
| 65 | 12.14 | | 4.17 | 0.245 |  | 13.64 | | 4.70 | 0.424 |  | 12.73 | | 3.82 | 0.161 |
| 70 | 12.02 | | 3.99 | 0.308 |  | 13.46 | | 4.48 | 0.478 |  | 12.48 | | 3.67 | 0.187 |
| 75 | 11.70 | | 3.74 | 0.370 |  | 13.04 | | 4.18 | 0.533 |  | 12.16 | | 3.49 | 0.213 |
| 80 | 11.27 | | 3.47 | 0.433 |  | 12.30 | | 3.80 | 0.587 |  | 11.79 | | 3.31 | 0.239 |
| 85 | 10.79 | | 3.19 | 0.495 |  | 11.46 | | 3.42 | 0.639 |  | 11.45 | | 3.15 | 0.262 |

M = Median, σ = Standard Deviation, L = Skewness (see LMS description in Methods).
