# Supplementary material for: Dual Energy X-Ray Absorptiometry Body Composition Reference Values from NHANES
Source: PLoS One. 2009 Sep 15;4(9):e7038. doi: 10.1371/journal.pone.0007038 (PMC2737140; doi:10.1371/journal.pone.0007038)
Supplement: Table S2 — %Fat (%) vs. Age in adult subjects. (0.08 MB DOC) [file pone.0007038.s022.doc]

Table S2: % Fat (%) vs. Age in adult subjects.

| **Males** | | | | | | | | | | | |
| --- | --- | --- | --- | --- | --- | --- | --- | --- | --- | --- | --- |
|  | White | | |  | Black | | |  | Mexican American | | |
| Age | M | σ | L |  | M | σ | L |  | M | σ | L |
| 20 | 23.4 | 6.68 | 0.221 |  | 19.8 | 6.60 | -0.311 |  | 24.4 | 6.09 | 0.075 |
| 25 | 24.6 | 6.49 | 0.325 |  | 21.7 | 6.89 | -0.123 |  | 26.0 | 5.68 | 0.238 |
| 30 | 25.7 | 6.25 | 0.428 |  | 23.1 | 6.92 | 0.064 |  | 27.2 | 5.26 | 0.400 |
| 35 | 26.6 | 5.97 | 0.530 |  | 24.2 | 6.75 | 0.250 |  | 27.9 | 5.00 | 0.561 |
| 40 | 27.5 | 5.68 | 0.631 |  | 25.0 | 6.52 | 0.434 |  | 28.4 | 4.93 | 0.720 |
| 45 | 28.2 | 5.44 | 0.732 |  | 25.6 | 6.33 | 0.617 |  | 28.8 | 4.96 | 0.877 |
| 50 | 29.0 | 5.30 | 0.831 |  | 26.1 | 6.23 | 0.799 |  | 29.2 | 5.02 | 1.033 |
| 55 | 29.8 | 5.23 | 0.930 |  | 26.9 | 6.22 | 0.980 |  | 29.8 | 5.03 | 1.188 |
| 60 | 30.5 | 5.19 | 1.028 |  | 27.7 | 6.22 | 1.160 |  | 30.2 | 4.99 | 1.343 |
| 65 | 31.1 | 5.16 | 1.126 |  | 28.6 | 6.14 | 1.339 |  | 30.6 | 4.88 | 1.497 |
| 70 | 31.4 | 5.11 | 1.223 |  | 29.3 | 5.97 | 1.519 |  | 30.7 | 4.73 | 1.652 |
| 75 | 31.6 | 5.02 | 1.320 |  | 30.0 | 5.68 | 1.698 |  | 30.7 | 4.55 | 1.806 |
| 80 | 31.6 | 4.88 | 1.418 |  | 30.5 | 5.29 | 1.877 |  | 30.7 | 4.36 | 1.960 |
| 85 | 31.6 | 4.73 | 1.515 |  | 31.0 | 4.89 | 2.045 |  | 30.7 | 4.18 | 2.114 |
| **Females** | | | | | | | | | | | |
|  | White | | |  | Black | | |  | Mexican American | | |
| Age | M | σ | L |  | M | σ | L |  | M | σ | L |
| 20 | 35.1 | 7.22 | 0.361 |  | 36.0 | 7.78 | 0.955 |  | 38.0 | 6.30 | 1.126 |
| 25 | 36.0 | 7.23 | 0.573 |  | 37.8 | 7.59 | 1.127 |  | 39.2 | 6.11 | 1.237 |
| 30 | 37.0 | 7.21 | 0.785 |  | 39.2 | 7.31 | 1.300 |  | 40.0 | 5.83 | 1.347 |
| 35 | 38.0 | 7.12 | 0.996 |  | 40.1 | 6.91 | 1.471 |  | 40.6 | 5.53 | 1.456 |
| 40 | 38.9 | 6.96 | 1.207 |  | 41.0 | 6.48 | 1.641 |  | 41.2 | 5.29 | 1.564 |
| 45 | 39.8 | 6.73 | 1.417 |  | 41.8 | 6.10 | 1.811 |  | 42.1 | 5.11 | 1.672 |
| 50 | 40.8 | 6.46 | 1.626 |  | 42.4 | 5.82 | 1.980 |  | 42.9 | 4.98 | 1.779 |
| 55 | 41.7 | 6.16 | 1.833 |  | 43.0 | 5.68 | 2.149 |  | 43.4 | 4.87 | 1.887 |
| 60 | 42.5 | 5.86 | 2.041 |  | 43.3 | 5.63 | 2.318 |  | 43.5 | 4.82 | 1.994 |
| 65 | 43.0 | 5.58 | 2.247 |  | 43.3 | 5.63 | 2.487 |  | 43.6 | 4.89 | 2.102 |
| 70 | 43.0 | 5.37 | 2.453 |  | 43.1 | 5.65 | 2.655 |  | 43.5 | 5.07 | 2.210 |
| 75 | 42.9 | 5.24 | 2.660 |  | 42.7 | 5.64 | 2.824 |  | 43.3 | 5.31 | 2.319 |
| 80 | 42.5 | 5.14 | 2.866 |  | 42.0 | 5.55 | 2.992 |  | 43.2 | 5.58 | 2.427 |
| 85 | 42.1 | 5.08 | 3.072 |  | 41.1 | 5.43 | 3.153 |  | 43.0 | 5.82 | 2.522 |

M = Median, σ = Standard Deviation, L = Skewness (see LMS description in Methods).
