# Supplementary material for: Dual Energy X-Ray Absorptiometry Body Composition Reference Values from NHANES
Source: PLoS One. 2009 Sep 15;4(9):e7038. doi: 10.1371/journal.pone.0007038 (PMC2737140; doi:10.1371/journal.pone.0007038)
Supplement: Table S3 — %Fat Trunk/%Fat Legs vs. Age in adult subjects. (0.08 MB DOC) [file pone.0007038.s023.doc]

Table S3: % Fat Trunk / % Fat Legs vs. Age in adult subjects.

| **Males** | | | | | | | | | | | | | | | |
| --- | --- | --- | --- | --- | --- | --- | --- | --- | --- | --- | --- | --- | --- | --- | --- |
|  | White | | |  | | Black | | | |  | Mexican American | | | | |
| Age | M | σ | L |  | | M | σ | | L |  | M | | σ | | L |
| 20 | 0.883 | 0.141 | 0.579 |  | | 0.844 | 0.141 | | 0.858 |  | 0.891 | | 0.133 | | 0.296 |
| 25 | 0.933 | 0.149 | 0.579 |  | | 0.887 | 0.148 | | 0.858 |  | 0.977 | | 0.146 | | 0.296 |
| 30 | 0.979 | 0.156 | 0.579 |  | | 0.928 | 0.155 | | 0.858 |  | 1.050 | | 0.156 | | 0.296 |
| 35 | 1.021 | 0.163 | 0.579 |  | | 0.965 | 0.161 | | 0.858 |  | 1.104 | | 0.164 | | 0.296 |
| 40 | 1.056 | 0.169 | 0.579 |  | | 0.995 | 0.166 | | 0.858 |  | 1.141 | | 0.170 | | 0.296 |
| 45 | 1.084 | 0.173 | 0.579 |  | | 1.017 | 0.170 | | 0.858 |  | 1.165 | | 0.173 | | 0.296 |
| 50 | 1.105 | 0.177 | 0.579 |  | | 1.037 | 0.173 | | 0.858 |  | 1.177 | | 0.175 | | 0.296 |
| 55 | 1.123 | 0.179 | 0.579 |  | | 1.057 | 0.177 | | 0.858 |  | 1.181 | | 0.176 | | 0.296 |
| 60 | 1.134 | 0.181 | 0.579 |  | | 1.068 | 0.178 | | 0.858 |  | 1.177 | | 0.175 | | 0.296 |
| 65 | 1.136 | 0.181 | 0.579 |  | | 1.066 | 0.178 | | 0.858 |  | 1.166 | | 0.174 | | 0.296 |
| 70 | 1.126 | 0.180 | 0.579 |  | | 1.056 | 0.176 | | 0.858 |  | 1.149 | | 0.171 | | 0.296 |
| 75 | 1.104 | 0.176 | 0.579 |  | | 1.042 | 0.174 | | 0.858 |  | 1.130 | | 0.168 | | 0.296 |
| 80 | 1.077 | 0.172 | 0.579 |  | | 1.026 | 0.171 | | 0.858 |  | 1.109 | | 0.165 | | 0.296 |
| 85 | 1.048 | 0.167 | 0.579 |  | | 1.012 | 0.169 | | 0.858 |  | 1.087 | | 0.162 | | 0.296 |
| **Females** | | | | | | | | | | | | | | | |
|  | White | | |  | Black | | | | |  | Mexican American | | | | |
| Age | M | σ | L |  | M | | σ | L | |  | M | σ | | L | |
| 20 | 0.759 | 0.161 | 0.658 |  | 0.847 | | 0.174 | 0.669 | |  | 0.874 | 0.149 | | 0.281 | |
| 25 | 0.786 | 0.164 | 0.658 |  | 0.865 | | 0.174 | 0.669 | |  | 0.883 | 0.147 | | 0.281 | |
| 30 | 0.810 | 0.166 | 0.658 |  | 0.880 | | 0.174 | 0.669 | |  | 0.895 | 0.147 | | 0.281 | |
| 35 | 0.830 | 0.167 | 0.658 |  | 0.892 | | 0.172 | 0.669 | |  | 0.911 | 0.147 | | 0.281 | |
| 40 | 0.848 | 0.167 | 0.658 |  | 0.902 | | 0.171 | 0.669 | |  | 0.928 | 0.147 | | 0.281 | |
| 45 | 0.863 | 0.167 | 0.658 |  | 0.913 | | 0.169 | 0.669 | |  | 0.945 | 0.147 | | 0.281 | |
| 50 | 0.878 | 0.167 | 0.658 |  | 0.922 | | 0.167 | 0.669 | |  | 0.959 | 0.146 | | 0.281 | |
| 55 | 0.892 | 0.166 | 0.658 |  | 0.929 | | 0.165 | 0.669 | |  | 0.968 | 0.145 | | 0.281 | |
| 60 | 0.902 | 0.164 | 0.658 |  | 0.933 | | 0.162 | 0.669 | |  | 0.972 | 0.143 | | 0.281 | |
| 65 | 0.906 | 0.162 | 0.658 |  | 0.932 | | 0.158 | 0.669 | |  | 0.970 | 0.140 | | 0.281 | |
| 70 | 0.904 | 0.158 | 0.658 |  | 0.926 | | 0.154 | 0.669 | |  | 0.962 | 0.136 | | 0.281 | |
| 75 | 0.896 | 0.153 | 0.658 |  | 0.916 | | 0.148 | 0.669 | |  | 0.948 | 0.132 | | 0.281 | |
| 80 | 0.885 | 0.148 | 0.658 |  | 0.902 | | 0.143 | 0.669 | |  | 0.931 | 0.127 | | 0.281 | |
| 85 | 0.872 | 0.142 | 0.658 |  | 0.888 | | 0.138 | 0.669 | |  | 0.916 | 0.123 | | 0.281 | |

M = Median, σ = Standard Deviation, L = Skewness (see LMS description in Methods).
