# Supplementary material for: Dual Energy X-Ray Absorptiometry Body Composition Reference Values from NHANES
Source: PLoS One. 2009 Sep 15;4(9):e7038. doi: 10.1371/journal.pone.0007038 (PMC2737140; doi:10.1371/journal.pone.0007038)
Supplement: Table S4 — Trunk to Limb Fat Mass Ratio vs. Age in Adult subjects. (0.08 MB DOC) [file pone.0007038.s024.doc]

Table S4: Trunk to Limb Fat Mass Ratio vs. Age in adult subjects.

| **Males** | | | | | | | | | | | | | | | | |
| --- | --- | --- | --- | --- | --- | --- | --- | --- | --- | --- | --- | --- | --- | --- | --- | --- |
|  | White | | |  | | Black | | | |  | | Mexican American | | | | |
| Age | M | σ | L |  | | M | σ | L | |  | | M | | σ | | L |
| 20 | 0.926 | 0.156 | 0.057 |  | | 0.776 | 0.136 | 0.076 | |  | | 0.939 | | 0.160 | | -0.468 |
| 25 | 0.995 | 0.179 | 0.121 |  | | 0.839 | 0.152 | 0.183 | |  | | 1.075 | | 0.196 | | -0.330 |
| 30 | 1.063 | 0.203 | 0.185 |  | | 0.898 | 0.171 | 0.290 | |  | | 1.190 | | 0.229 | | -0.192 |
| 35 | 1.125 | 0.224 | 0.249 |  | | 0.951 | 0.189 | 0.398 | |  | | 1.274 | | 0.249 | | -0.053 |
| 40 | 1.183 | 0.241 | 0.312 |  | | 0.999 | 0.204 | 0.505 | |  | | 1.331 | | 0.255 | | 0.086 |
| 45 | 1.236 | 0.254 | 0.375 |  | | 1.040 | 0.214 | 0.612 | |  | | 1.372 | | 0.254 | | 0.223 |
| 50 | 1.281 | 0.261 | 0.439 |  | | 1.078 | 0.222 | 0.720 | |  | | 1.406 | | 0.253 | | 0.359 |
| 55 | 1.321 | 0.265 | 0.502 |  | | 1.114 | 0.232 | 0.827 | |  | | 1.432 | | 0.252 | | 0.493 |
| 60 | 1.351 | 0.267 | 0.565 |  | | 1.140 | 0.241 | 0.934 | |  | | 1.447 | | 0.249 | | 0.627 |
| 65 | 1.366 | 0.268 | 0.629 |  | | 1.151 | 0.246 | 1.042 | |  | | 1.449 | | 0.243 | | 0.760 |
| 70 | 1.361 | 0.267 | 0.692 |  | | 1.150 | 0.248 | 1.149 | |  | | 1.438 | | 0.236 | | 0.893 |
| 75 | 1.339 | 0.263 | 0.755 |  | | 1.139 | 0.246 | 1.256 | |  | | 1.423 | | 0.229 | | 1.027 |
| 80 | 1.306 | 0.257 | 0.819 |  | | 1.124 | 0.241 | 1.364 | |  | | 1.406 | | 0.223 | | 1.160 |
| 85 | 1.271 | 0.250 | 0.882 |  | | 1.108 | 0.236 | 1.464 | |  | | 1.387 | | 0.217 | | 1.293 |
| **Females** | | | | | | | | | | | | | | | | |
|  | White | | |  | Black | | | |  | | Mexican American | | | | | |
| Age | M | σ | L |  | M | | σ | L |  | | M | | σ | | L | |
| 20 | 0.745 | 0.192 | 0.183 |  | 0.762 | | 0.204 | 0.346 |  | | 0.961 | | 0.226 | | 0.074 | |
| 25 | 0.796 | 0.207 | 0.183 |  | 0.797 | | 0.212 | 0.346 |  | | 0.965 | | 0.221 | | 0.074 | |
| 30 | 0.841 | 0.220 | 0.183 |  | 0.823 | | 0.217 | 0.346 |  | | 0.983 | | 0.219 | | 0.074 | |
| 35 | 0.873 | 0.228 | 0.183 |  | 0.842 | | 0.219 | 0.346 |  | | 1.018 | | 0.222 | | 0.074 | |
| 40 | 0.897 | 0.235 | 0.183 |  | 0.865 | | 0.222 | 0.346 |  | | 1.054 | | 0.227 | | 0.074 | |
| 45 | 0.920 | 0.240 | 0.183 |  | 0.890 | | 0.224 | 0.346 |  | | 1.092 | | 0.232 | | 0.074 | |
| 50 | 0.947 | 0.245 | 0.183 |  | 0.914 | | 0.227 | 0.346 |  | | 1.127 | | 0.239 | | 0.074 | |
| 55 | 0.975 | 0.251 | 0.183 |  | 0.929 | | 0.230 | 0.346 |  | | 1.155 | | 0.246 | | 0.074 | |
| 60 | 0.998 | 0.255 | 0.183 |  | 0.940 | | 0.233 | 0.346 |  | | 1.169 | | 0.253 | | 0.074 | |
| 65 | 1.011 | 0.257 | 0.183 |  | 0.945 | | 0.236 | 0.346 |  | | 1.171 | | 0.260 | | 0.074 | |
| 70 | 1.011 | 0.256 | 0.183 |  | 0.939 | | 0.237 | 0.346 |  | | 1.160 | | 0.265 | | 0.074 | |
| 75 | 0.999 | 0.252 | 0.183 |  | 0.923 | | 0.238 | 0.346 |  | | 1.134 | | 0.268 | | 0.074 | |
| 80 | 0.976 | 0.247 | 0.183 |  | 0.903 | | 0.238 | 0.346 |  | | 1.098 | | 0.270 | | 0.074 | |
| 85 | 0.949 | 0.240 | 0.183 |  | 0.882 | | 0.238 | 0.346 |  | | 1.065 | | 0.270 | | 0.074 | |

M = Median, σ = Standard Deviation, L = Skewness (see LMS description in Methods).
