# Supplementary material for: Dual Energy X-Ray Absorptiometry Body Composition Reference Values from NHANES
Source: PLoS One. 2009 Sep 15;4(9):e7038. doi: 10.1371/journal.pone.0007038 (PMC2737140; doi:10.1371/journal.pone.0007038)
Supplement: Table S5 — Lean Mass/Height2 (kg/m2) vs. Age in adult subjects. (0.08 MB DOC) [file pone.0007038.s025.doc]

Table S5: Lean Mass/Height^2^ (kg/m^2^) vs. Age in adult subjects.

| **Males** | | | | | | | | | | | | |
| --- | --- | --- | --- | --- | --- | --- | --- | --- | --- | --- | --- | --- |
|  | White | | |  | Black | | |  | Mexican American | | | |
| Age | M | σ | L |  | M | σ | L |  | M | | σ | L |
| 20 | 18.98 | 2.50 | -1.115 |  | 19.50 | 2.98 | -1.103 |  | 18.78 | | 2.22 | -0.738 |
| 25 | 19.31 | 2.52 | -1.022 |  | 19.97 | 3.01 | -0.969 |  | 19.36 | | 2.23 | -0.703 |
| 30 | 19.60 | 2.54 | -0.929 |  | 20.33 | 3.01 | -0.835 |  | 19.83 | | 2.24 | -0.667 |
| 35 | 19.85 | 2.56 | -0.835 |  | 20.54 | 2.98 | -0.701 |  | 20.16 | | 2.25 | -0.633 |
| 40 | 20.04 | 2.56 | -0.740 |  | 20.60 | 2.93 | -0.567 |  | 20.34 | | 2.26 | -0.600 |
| 45 | 20.15 | 2.55 | -0.643 |  | 20.56 | 2.88 | -0.434 |  | 20.37 | | 2.26 | -0.571 |
| 50 | 20.15 | 2.53 | -0.546 |  | 20.47 | 2.85 | -0.301 |  | 20.31 | | 2.25 | -0.543 |
| 55 | 20.07 | 2.50 | -0.447 |  | 20.35 | 2.83 | -0.169 |  | 20.15 | | 2.21 | -0.517 |
| 60 | 19.91 | 2.44 | -0.348 |  | 20.19 | 2.80 | -0.037 |  | 19.93 | | 2.16 | -0.492 |
| 65 | 19.67 | 2.36 | -0.248 |  | 19.95 | 2.77 | 0.096 |  | 19.63 | | 2.10 | -0.467 |
| 70 | 19.36 | 2.26 | -0.148 |  | 19.65 | 2.71 | 0.228 |  | 19.26 | | 2.02 | -0.442 |
| 75 | 18.98 | 2.15 | -0.047 |  | 19.30 | 2.65 | 0.360 |  | 18.84 | | 1.94 | -0.418 |
| 80 | 18.58 | 2.03 | 0.053 |  | 18.93 | 2.58 | 0.493 |  | 18.39 | | 1.85 | -0.393 |
| 85 | 18.16 | 1.92 | 0.154 |  | 18.59 | 2.51 | 0.616 |  | 17.93 | | 1.76 | -0.369 |
| **Females** | | | | | | | | | | | | |
|  | White | | |  | Black | | |  | | Mexican American | | |
| Age | M | σ | L |  | M | σ | L |  | | M | σ | L |
| 20 | 15.60 | 2.01 | -1.404 |  | 17.24 | 2.74 | -0.924 |  | | 15.82 | 2.08 | -1.510 |
| 25 | 15.83 | 2.10 | -1.378 |  | 17.60 | 2.80 | -0.911 |  | | 16.24 | 2.18 | -1.411 |
| 30 | 16.03 | 2.18 | -1.352 |  | 17.87 | 2.84 | -0.898 |  | | 16.58 | 2.28 | -1.311 |
| 35 | 16.19 | 2.26 | -1.326 |  | 18.03 | 2.86 | -0.885 |  | | 16.83 | 2.36 | -1.212 |
| 40 | 16.30 | 2.32 | -1.299 |  | 18.12 | 2.86 | -0.872 |  | | 17.02 | 2.42 | -1.112 |
| 45 | 16.36 | 2.35 | -1.272 |  | 18.13 | 2.84 | -0.859 |  | | 17.14 | 2.45 | -1.013 |
| 50 | 16.35 | 2.36 | -1.244 |  | 18.07 | 2.80 | -0.844 |  | | 17.13 | 2.46 | -0.912 |
| 55 | 16.30 | 2.35 | -1.216 |  | 17.97 | 2.73 | -0.830 |  | | 17.00 | 2.43 | -0.811 |
| 60 | 16.21 | 2.30 | -1.188 |  | 17.88 | 2.66 | -0.816 |  | | 16.81 | 2.39 | -0.710 |
| 65 | 16.08 | 2.24 | -1.160 |  | 17.77 | 2.56 | -0.803 |  | | 16.57 | 2.35 | -0.607 |
| 70 | 15.92 | 2.16 | -1.132 |  | 17.61 | 2.46 | -0.789 |  | | 16.28 | 2.30 | -0.505 |
| 75 | 15.73 | 2.07 | -1.104 |  | 17.39 | 2.35 | -0.776 |  | | 15.97 | 2.25 | -0.402 |
| 80 | 15.53 | 1.98 | -1.076 |  | 17.12 | 2.24 | -0.763 |  | | 15.63 | 2.19 | -0.299 |
| 85 | 15.32 | 1.89 | -1.049 |  | 16.85 | 2.13 | -0.750 |  | | 15.34 | 2.14 | -0.210 |

M = Median, σ = Standard Deviation, L = Skewness (see LMS description in Methods).
