# Supplementary material for: Dual Energy X-Ray Absorptiometry Body Composition Reference Values from NHANES
Source: PLoS One. 2009 Sep 15;4(9):e7038. doi: 10.1371/journal.pone.0007038 (PMC2737140; doi:10.1371/journal.pone.0007038)
Supplement: Table S6 — Appendicular Lean Mass/Height2 (kg/m2) vs. Age in adult subjects. (0.08 MB DOC) [file pone.0007038.s026.doc]

Table S6: Appendicular Lean Mass/Height^2^ (kg/m^2^) vs. Age in adult subjects.

| **Males** | | | | | | | | | | | |
| --- | --- | --- | --- | --- | --- | --- | --- | --- | --- | --- | --- |
|  | White | | |  | Black | | |  | Mexican American | | |
| Age | M | σ | L |  | M | σ | L |  | M | σ | L |
| 20 | 8.87 | 1.34 | -0.708 |  | 9.68 | 1.78 | -0.561 |  | 8.56 | 1.05 | -0.940 |
| 25 | 8.94 | 1.32 | -0.616 |  | 9.81 | 1.73 | -0.460 |  | 8.76 | 1.07 | -0.795 |
| 30 | 9.02 | 1.31 | -0.524 |  | 9.91 | 1.68 | -0.359 |  | 8.93 | 1.08 | -0.650 |
| 35 | 9.09 | 1.30 | -0.432 |  | 9.94 | 1.62 | -0.258 |  | 9.05 | 1.09 | -0.507 |
| 40 | 9.12 | 1.29 | -0.341 |  | 9.90 | 1.56 | -0.157 |  | 9.11 | 1.11 | -0.368 |
| 45 | 9.11 | 1.27 | -0.249 |  | 9.79 | 1.50 | -0.057 |  | 9.09 | 1.11 | -0.233 |
| 50 | 9.05 | 1.25 | -0.157 |  | 9.67 | 1.47 | 0.042 |  | 9.02 | 1.11 | -0.101 |
| 55 | 8.95 | 1.22 | -0.065 |  | 9.55 | 1.44 | 0.140 |  | 8.89 | 1.09 | 0.029 |
| 60 | 8.81 | 1.18 | 0.027 |  | 9.40 | 1.43 | 0.238 |  | 8.72 | 1.06 | 0.157 |
| 65 | 8.64 | 1.14 | 0.118 |  | 9.22 | 1.40 | 0.334 |  | 8.52 | 1.03 | 0.284 |
| 70 | 8.44 | 1.09 | 0.210 |  | 9.01 | 1.38 | 0.431 |  | 8.28 | 0.98 | 0.410 |
| 75 | 8.21 | 1.03 | 0.302 |  | 8.78 | 1.34 | 0.528 |  | 8.01 | 0.93 | 0.536 |
| 80 | 7.97 | 0.97 | 0.394 |  | 8.56 | 1.31 | 0.624 |  | 7.73 | 0.88 | 0.662 |
| 85 | 7.72 | 0.91 | 0.486 |  | 8.34 | 1.28 | 0.714 |  | 7.44 | 0.83 | 0.788 |
| **Females** | | | | | | | | | | | |
|  | White | | |  | Black | | |  | Mexican American | | |
| Age | M | σ | L |  | M | σ | L |  | M | σ | L |
| 20 | 6.81 | 1.04 | -0.818 |  | 8.23 | 1.47 | -0.507 |  | 6.72 | 1.01 | -1.307 |
| 25 | 6.86 | 1.08 | -0.818 |  | 8.29 | 1.49 | -0.522 |  | 6.84 | 1.06 | -1.169 |
| 30 | 6.90 | 1.11 | -0.817 |  | 8.32 | 1.51 | -0.537 |  | 6.95 | 1.11 | -1.032 |
| 35 | 6.93 | 1.15 | -0.817 |  | 8.32 | 1.52 | -0.551 |  | 7.03 | 1.15 | -0.893 |
| 40 | 6.95 | 1.17 | -0.816 |  | 8.29 | 1.51 | -0.565 |  | 7.09 | 1.18 | -0.755 |
| 45 | 6.93 | 1.19 | -0.815 |  | 8.22 | 1.49 | -0.579 |  | 7.11 | 1.20 | -0.617 |
| 50 | 6.90 | 1.18 | -0.813 |  | 8.13 | 1.47 | -0.592 |  | 7.06 | 1.20 | -0.479 |
| 55 | 6.84 | 1.17 | -0.811 |  | 8.03 | 1.43 | -0.604 |  | 6.96 | 1.18 | -0.340 |
| 60 | 6.76 | 1.14 | -0.810 |  | 7.94 | 1.39 | -0.617 |  | 6.83 | 1.16 | -0.202 |
| 65 | 6.67 | 1.10 | -0.809 |  | 7.85 | 1.35 | -0.629 |  | 6.69 | 1.13 | -0.065 |
| 70 | 6.57 | 1.06 | -0.808 |  | 7.73 | 1.30 | -0.642 |  | 6.54 | 1.10 | 0.073 |
| 75 | 6.45 | 1.01 | -0.808 |  | 7.59 | 1.24 | -0.654 |  | 6.37 | 1.06 | 0.211 |
| 80 | 6.33 | 0.96 | -0.808 |  | 7.41 | 1.19 | -0.666 |  | 6.20 | 1.03 | 0.348 |
| 85 | 6.20 | 0.91 | -0.807 |  | 7.22 | 1.13 | -0.678 |  | 6.04 | 0.99 | 0.468 |

M = Median, σ = Standard Deviation, L = Skewness (see LMS description in Methods).
