# Supplementary material for: Dual Energy X-Ray Absorptiometry Body Composition Reference Values from NHANES
Source: PLoS One. 2009 Sep 15;4(9):e7038. doi: 10.1371/journal.pone.0007038 (PMC2737140; doi:10.1371/journal.pone.0007038)
Supplement: Table S7 — Total Body BMD (g/cm2) vs. Age in adult subjects. (0.08 MB DOC) [file pone.0007038.s027.doc]

Table S7: Total Body BMD (g/cm^2^) vs. Age in adult subjects.

| **Males** | | | | | | | | | | | |
| --- | --- | --- | --- | --- | --- | --- | --- | --- | --- | --- | --- |
|  | White | | |  | Black | | |  | Mexican American | | |
| Age | M | σ | L |  | M | σ | L |  | M | σ | L |
| 20 | 1.185 | 0.097 | 0.026 |  | 1.262 | 0.114 | -0.436 |  | 1.144 | 0.093 | -0.205 |
| 25 | 1.196 | 0.100 | 0.026 |  | 1.281 | 0.117 | -0.436 |  | 1.156 | 0.095 | -0.205 |
| 30 | 1.202 | 0.102 | 0.026 |  | 1.285 | 0.119 | -0.436 |  | 1.158 | 0.097 | -0.205 |
| 35 | 1.202 | 0.104 | 0.026 |  | 1.274 | 0.119 | -0.436 |  | 1.155 | 0.098 | -0.205 |
| 40 | 1.198 | 0.106 | 0.026 |  | 1.257 | 0.119 | -0.436 |  | 1.151 | 0.100 | -0.205 |
| 45 | 1.192 | 0.107 | 0.026 |  | 1.243 | 0.119 | -0.436 |  | 1.144 | 0.101 | -0.205 |
| 50 | 1.185 | 0.108 | 0.026 |  | 1.236 | 0.119 | -0.436 |  | 1.136 | 0.102 | -0.205 |
| 55 | 1.175 | 0.109 | 0.026 |  | 1.233 | 0.120 | -0.436 |  | 1.130 | 0.103 | -0.205 |
| 60 | 1.164 | 0.110 | 0.026 |  | 1.228 | 0.121 | -0.436 |  | 1.124 | 0.104 | -0.205 |
| 65 | 1.155 | 0.111 | 0.026 |  | 1.216 | 0.121 | -0.436 |  | 1.117 | 0.105 | -0.205 |
| 70 | 1.144 | 0.111 | 0.026 |  | 1.196 | 0.120 | -0.436 |  | 1.110 | 0.106 | -0.205 |
| 75 | 1.128 | 0.111 | 0.026 |  | 1.170 | 0.119 | -0.436 |  | 1.101 | 0.107 | -0.205 |
| 80 | 1.111 | 0.111 | 0.026 |  | 1.143 | 0.117 | -0.436 |  | 1.092 | 0.107 | -0.205 |
| 85 | 1.093 | 0.111 | 0.026 |  | 1.118 | 0.115 | -0.436 |  | 1.082 | 0.108 | -0.205 |
| **Females** | | | | | | | | | | | |
|  | White | | |  | Black | | |  | Mexican American | | |
| Age | M | σ | L |  | M | σ | L |  | M | σ | L |
| 20 | 1.094 | 0.076 | 0.319 |  | 1.171 | 0.087 | 0.185 |  | 1.084 | 0.076 | 0.667 |
| 25 | 1.106 | 0.081 | 0.319 |  | 1.181 | 0.091 | 0.185 |  | 1.097 | 0.081 | 0.667 |
| 30 | 1.115 | 0.085 | 0.319 |  | 1.189 | 0.095 | 0.185 |  | 1.106 | 0.085 | 0.667 |
| 35 | 1.121 | 0.089 | 0.319 |  | 1.191 | 0.099 | 0.185 |  | 1.110 | 0.090 | 0.667 |
| 40 | 1.122 | 0.092 | 0.319 |  | 1.188 | 0.102 | 0.185 |  | 1.107 | 0.093 | 0.667 |
| 45 | 1.116 | 0.095 | 0.319 |  | 1.177 | 0.105 | 0.185 |  | 1.096 | 0.096 | 0.667 |
| 50 | 1.102 | 0.098 | 0.319 |  | 1.159 | 0.107 | 0.185 |  | 1.077 | 0.098 | 0.667 |
| 55 | 1.083 | 0.099 | 0.319 |  | 1.137 | 0.109 | 0.185 |  | 1.053 | 0.100 | 0.667 |
| 60 | 1.059 | 0.101 | 0.319 |  | 1.111 | 0.110 | 0.185 |  | 1.025 | 0.101 | 0.667 |
| 65 | 1.033 | 0.102 | 0.319 |  | 1.084 | 0.110 | 0.185 |  | 0.996 | 0.101 | 0.667 |
| 70 | 1.006 | 0.102 | 0.319 |  | 1.056 | 0.111 | 0.185 |  | 0.966 | 0.102 | 0.667 |
| 75 | 0.977 | 0.102 | 0.319 |  | 1.028 | 0.111 | 0.185 |  | 0.935 | 0.102 | 0.667 |
| 80 | 0.948 | 0.102 | 0.319 |  | 0.999 | 0.111 | 0.185 |  | 0.905 | 0.102 | 0.667 |
| 85 | 0.920 | 0.102 | 0.319 |  | 0.971 | 0.111 | 0.185 |  | 0.880 | 0.101 | 0.667 |

M = Median, σ = Standard Deviation, L = Skewness (see LMS description in Methods).
