# Supplementary material for: Dual Energy X-Ray Absorptiometry Body Composition Reference Values from NHANES
Source: PLoS One. 2009 Sep 15;4(9):e7038. doi: 10.1371/journal.pone.0007038 (PMC2737140; doi:10.1371/journal.pone.0007038)
Supplement: Table S8 — Total Body BMC (g) vs. Age in adult subjects. (0.08 MB DOC) [file pone.0007038.s028.doc]

Table S8: Total Body BMC (g) vs. Age in adult subjects.

| **Males** | | | | | | | | | | | |
| --- | --- | --- | --- | --- | --- | --- | --- | --- | --- | --- | --- |
|  | White | | |  | Black | | |  | Mexican American | | |
| Age | M | σ | L |  | M | σ | L |  | M | σ | L |
| 20 | 2705 | 431 | -0.284 |  | 2951 | 498 | -0.299 |  | 2455 | 385 | -0.360 |
| 25 | 2734 | 430 | -0.208 |  | 2991 | 490 | -0.223 |  | 2470 | 377 | -0.282 |
| 30 | 2755 | 427 | -0.132 |  | 3005 | 483 | -0.147 |  | 2474 | 365 | -0.202 |
| 35 | 2766 | 420 | -0.055 |  | 2990 | 476 | -0.070 |  | 2472 | 356 | -0.122 |
| 40 | 2768 | 411 | 0.021 |  | 2955 | 469 | 0.009 |  | 2466 | 357 | -0.041 |
| 45 | 2759 | 403 | 0.097 |  | 2915 | 462 | 0.088 |  | 2455 | 362 | 0.041 |
| 50 | 2739 | 402 | 0.174 |  | 2883 | 453 | 0.167 |  | 2441 | 365 | 0.124 |
| 55 | 2709 | 406 | 0.250 |  | 2858 | 445 | 0.247 |  | 2426 | 366 | 0.208 |
| 60 | 2673 | 411 | 0.327 |  | 2829 | 439 | 0.326 |  | 2408 | 367 | 0.294 |
| 65 | 2632 | 414 | 0.403 |  | 2785 | 437 | 0.405 |  | 2384 | 368 | 0.380 |
| 70 | 2583 | 415 | 0.479 |  | 2727 | 438 | 0.484 |  | 2354 | 368 | 0.466 |
| 75 | 2524 | 414 | 0.556 |  | 2657 | 442 | 0.562 |  | 2319 | 368 | 0.553 |
| 80 | 2459 | 412 | 0.632 |  | 2586 | 448 | 0.641 |  | 2283 | 368 | 0.639 |
| 85 | 2393 | 409 | 0.708 |  | 2520 | 455 | 0.714 |  | 2246 | 367 | 0.726 |
| **Females** | | | | | | | | | | | |
|  | White | | |  | Black | | |  | Mexican American | | |
| Age | M | σ | L |  | M | σ | L |  | M | σ | L |
| 20 | 2124 | 286 | 0.138 |  | 2322 | 326 | -0.179 |  | 2011 | 282 | -0.017 |
| 25 | 2152 | 289 | 0.171 |  | 2360 | 335 | -0.109 |  | 2043 | 272 | 0.092 |
| 30 | 2177 | 294 | 0.204 |  | 2390 | 340 | -0.039 |  | 2065 | 269 | 0.201 |
| 35 | 2194 | 301 | 0.237 |  | 2406 | 342 | 0.031 |  | 2072 | 272 | 0.310 |
| 40 | 2198 | 306 | 0.271 |  | 2402 | 343 | 0.103 |  | 2067 | 278 | 0.419 |
| 45 | 2184 | 308 | 0.306 |  | 2378 | 341 | 0.175 |  | 2044 | 286 | 0.527 |
| 50 | 2150 | 309 | 0.342 |  | 2334 | 340 | 0.250 |  | 2001 | 296 | 0.635 |
| 55 | 2099 | 311 | 0.379 |  | 2278 | 342 | 0.324 |  | 1940 | 305 | 0.743 |
| 60 | 2034 | 314 | 0.417 |  | 2211 | 343 | 0.399 |  | 1867 | 312 | 0.850 |
| 65 | 1960 | 316 | 0.456 |  | 2134 | 344 | 0.474 |  | 1786 | 316 | 0.957 |
| 70 | 1879 | 316 | 0.495 |  | 2049 | 344 | 0.549 |  | 1701 | 318 | 1.063 |
| 75 | 1793 | 312 | 0.534 |  | 1958 | 341 | 0.624 |  | 1614 | 317 | 1.170 |
| 80 | 1703 | 306 | 0.574 |  | 1864 | 336 | 0.699 |  | 1528 | 314 | 1.276 |
| 85 | 1612 | 299 | 0.613 |  | 1773 | 330 | 0.770 |  | 1453 | 310 | 1.368 |

M = Median, σ = Standard Deviation, L = Skewness (see LMS description in Methods).
