# Supplementary material for: Dual Energy X-Ray Absorptiometry Body Composition Reference Values from NHANES
Source: PLoS One. 2009 Sep 15;4(9):e7038. doi: 10.1371/journal.pone.0007038 (PMC2737140; doi:10.1371/journal.pone.0007038)
Supplement: Table S9 — %Fat (%) vs. Age in pediatric subjects. (0.05 MB DOC) [file pone.0007038.s029.doc]

Table S9: % Fat (%) vs. Age in pediatric subjects.

| **Males** | | | | | | | | | | | |
| --- | --- | --- | --- | --- | --- | --- | --- | --- | --- | --- | --- |
|  | White | | |  | Black | | |  | Mexican American | | |
| Age | M | σ | L |  | M | σ | L |  | M | σ | L |
| 8 | 26.5 | 7.13 | -0.633 |  | 23.8 | 7.18 | -0.862 |  | 28.7 | 8.07 | -0.045 |
| 10 | 27.1 | 7.28 | -0.633 |  | 23.5 | 7.07 | -0.862 |  | 30.0 | 8.44 | -0.045 |
| 12 | 26.4 | 7.11 | -0.633 |  | 22.8 | 6.88 | -0.862 |  | 29.0 | 8.17 | -0.045 |
| 14 | 23.8 | 6.40 | -0.633 |  | 20.9 | 6.31 | -0.862 |  | 25.7 | 7.22 | -0.045 |
| 16 | 21.3 | 5.72 | -0.633 |  | 19.0 | 5.71 | -0.862 |  | 24.0 | 6.76 | -0.045 |
| 18 | 22.1 | 6.22 | -0.227 |  | 18.7 | 5.98 | -0.624 |  | 23.7 | 6.44 | -0.018 |
| 20 | 23.4 | 6.68 | 0.221 |  | 19.8 | 6.60 | -0.311 |  | 24.4 | 6.09 | 0.075 |
| **Females** | | | | | | | | | | | |
|  | White | | |  | Black | | |  | Mexican American | | |
| Age | M | σ | L |  | M | σ | L |  | M | σ | L |
| 8 | 31.6 | 6.50 | -0.041 |  | 29.3 | 7.03 | 0.236 |  | 31.2 | 5.93 | 0.156 |
| 10 | 31.7 | 6.52 | -0.041 |  | 30.3 | 7.28 | 0.236 |  | 32.4 | 6.15 | 0.156 |
| 12 | 31.4 | 6.46 | -0.041 |  | 30.6 | 7.35 | 0.236 |  | 32.8 | 6.24 | 0.156 |
| 14 | 31.5 | 6.49 | -0.041 |  | 31.2 | 7.47 | 0.236 |  | 33.5 | 6.37 | 0.156 |
| 16 | 32.5 | 6.68 | -0.041 |  | 32.5 | 7.79 | 0.236 |  | 34.6 | 6.57 | 0.156 |
| 18 | 34.3 | 7.10 | 0.118 |  | 34.5 | 7.95 | 0.561 |  | 36.8 | 6.60 | 0.619 |
| 20 | 35.1 | 7.22 | 0.361 |  | 36.0 | 7.78 | 0.955 |  | 38.0 | 6.30 | 1.126 |

M = Median, σ = Standard Deviation, L = Skewness (see LMS description in Methods).
