# Supplementary material for: Dual Energy X-Ray Absorptiometry Body Composition Reference Values from NHANES
Source: PLoS One. 2009 Sep 15;4(9):e7038. doi: 10.1371/journal.pone.0007038 (PMC2737140; doi:10.1371/journal.pone.0007038)
Supplement: Table S10 — Lean Mass/Height2 (kg/m2) vs. Age in pediatric subjects. (0.05 MB DOC) [file pone.0007038.s030.doc]

Table S10: Lean Mass/Height^2^ (kg/m^2^) vs. Age in pediatric subjects.

| **Males** | | | | | | | | | | | | |
| --- | --- | --- | --- | --- | --- | --- | --- | --- | --- | --- | --- | --- |
|  | White | | |  | Black | | |  | Mexican American | | | |
| Age | M | σ | L |  | M | σ | L |  | M | | σ | L |
| 8 | 12.91 | 1.37 | -0.294 |  | 13.10 | 1.23 | -0.736 |  | 12.72 | | 1.46 | -1.072 |
| 10 | 13.25 | 1.46 | -0.437 |  | 13.88 | 1.59 | -0.827 |  | 13.53 | | 1.74 | -0.897 |
| 12 | 14.04 | 1.65 | -0.577 |  | 14.91 | 1.97 | -0.919 |  | 14.57 | | 2.01 | -0.725 |
| 14 | 15.69 | 1.94 | -0.715 |  | 16.56 | 2.36 | -1.011 |  | 16.18 | | 2.27 | -0.560 |
| 16 | 17.43 | 2.17 | -0.849 |  | 18.21 | 2.64 | -1.103 |  | 17.80 | | 2.47 | -0.400 |
| 18 | 18.68 | 2.39 | -1.067 |  | 19.18 | 2.84 | -1.176 |  | 18.53 | | 2.38 | -0.496 |
| 20 | 18.98 | 2.50 | -1.115 |  | 19.50 | 2.98 | -1.103 |  | 18.78 | | 2.22 | -0.738 |
| **Females** | | | | | | | | | | | | |
|  | White | | |  | Black | | |  | | Mexican American | | |
| Age | M | σ | L |  | M | σ | L |  | | M | σ | L |
| 8 | 11.72 | 1.40 | -0.670 |  | 12.38 | 1.62 | -0.906 |  | | 11.56 | 1.30 | -1.443 |
| 10 | 12.83 | 1.54 | -0.761 |  | 13.77 | 1.87 | -1.007 |  | | 12.56 | 1.53 | -1.493 |
| 12 | 13.82 | 1.67 | -0.855 |  | 15.00 | 2.08 | -1.112 |  | | 13.92 | 1.77 | -1.542 |
| 14 | 14.58 | 1.75 | -0.950 |  | 15.89 | 2.20 | -1.219 |  | | 14.71 | 1.90 | -1.587 |
| 16 | 15.00 | 1.81 | -1.045 |  | 16.26 | 2.33 | -1.326 |  | | 14.89 | 1.94 | -1.626 |
| 18 | 15.40 | 1.93 | -1.277 |  | 16.77 | 2.60 | -1.181 |  | | 15.46 | 2.02 | -1.605 |
| 20 | 15.60 | 2.01 | -1.404 |  | 17.24 | 2.74 | -0.924 |  | | 15.82 | 2.08 | -1.510 |

M = Median, σ = Standard Deviation, L = Skewness (see LMS description in Methods).
