# Supplementary material for: Dual Energy X-Ray Absorptiometry Body Composition Reference Values from NHANES
Source: PLoS One. 2009 Sep 15;4(9):e7038. doi: 10.1371/journal.pone.0007038 (PMC2737140; doi:10.1371/journal.pone.0007038)
Supplement: Table S11 — Total Body BMD (g/cm2) vs. Age in pediatric subjects. (0.05 MB DOC) [file pone.0007038.s031.doc]

Table S11: Total Body BMD (g/cm^2^) vs. Age in pediatric subjects.

| **Males** | | | | | | | | | | | |
| --- | --- | --- | --- | --- | --- | --- | --- | --- | --- | --- | --- |
|  | White | | |  | Black | | |  | Mexican American | | |
| Age | M | σ | L |  | M | σ | L |  | M | σ | L |
| 8 | 0.782 | 0.057 | 1.889 |  | 0.817 | 0.067 | 0.586 |  | 0.760 | 0.058 | 0.344 |
| 10 | 0.832 | 0.059 | 0.952 |  | 0.877 | 0.067 | 0.604 |  | 0.823 | 0.065 | 0.046 |
| 12 | 0.885 | 0.068 | 0.264 |  | 0.937 | 0.074 | 0.455 |  | 0.886 | 0.073 | -0.056 |
| 14 | 0.978 | 0.084 | 0.069 |  | 1.035 | 0.094 | 0.312 |  | 0.978 | 0.085 | 0.003 |
| 16 | 1.094 | 0.098 | 0.318 |  | 1.155 | 0.110 | 0.378 |  | 1.084 | 0.097 | 0.043 |
| 18 | 1.174 | 0.098 | 0.423 |  | 1.241 | 0.112 | 0.039 |  | 1.137 | 0.096 | -0.112 |
| 20 | 1.185 | 0.097 | 0.026 |  | 1.262 | 0.114 | -0.436 |  | 1.144 | 0.093 | -0.205 |
| **Females** | | | | | | | | | | | |
|  | White | | |  | Black | | |  | Mexican American | | |
| Age | M | σ | L |  | M | σ | L |  | M | σ | L |
| 8 | 0.742 | 0.056 | 0.798 |  | 0.778 | 0.059 | 1.635 |  | 0.718 | 0.057 | -0.097 |
| 10 | 0.823 | 0.065 | 0.832 |  | 0.876 | 0.069 | 1.283 |  | 0.812 | 0.065 | 0.058 |
| 12 | 0.913 | 0.074 | 0.866 |  | 0.985 | 0.080 | 0.935 |  | 0.921 | 0.075 | 0.209 |
| 14 | 0.999 | 0.080 | 0.901 |  | 1.082 | 0.088 | 0.599 |  | 1.007 | 0.082 | 0.357 |
| 16 | 1.058 | 0.083 | 0.935 |  | 1.130 | 0.091 | 0.281 |  | 1.056 | 0.083 | 0.507 |
| 18 | 1.089 | 0.079 | 0.644 |  | 1.160 | 0.088 | 0.079 |  | 1.077 | 0.079 | 0.664 |
| 20 | 1.094 | 0.076 | 0.319 |  | 1.171 | 0.087 | 0.185 |  | 1.084 | 0.076 | 0.667 |

M = Median, σ = Standard Deviation, L = Skewness (see LMS description in Methods).
