# Supplementary material for: Dual Energy X-Ray Absorptiometry Body Composition Reference Values from NHANES
Source: PLoS One. 2009 Sep 15;4(9):e7038. doi: 10.1371/journal.pone.0007038 (PMC2737140; doi:10.1371/journal.pone.0007038)
Supplement: Table S12 — Total Body BMC (g) vs. Age in Pediatric subjects. (0.05 MB DOC) [file pone.0007038.s032.doc]

Table S12: Total Body BMC (g) vs. Age in pediatric subjects.

| **Males** | | | | | | | | | | | |
| --- | --- | --- | --- | --- | --- | --- | --- | --- | --- | --- | --- |
|  | White | | |  | Black | | |  | Mexican American | | |
| Age | M | σ | L |  | M | σ | L |  | M | σ | L |
| 8 | 951 | 112 | 0.781 |  | 1033 | 135 | -0.674 |  | 934 | 110 | 0.097 |
| 10 | 1123 | 163 | 0.316 |  | 1235 | 184 | -0.357 |  | 1128 | 169 | -0.037 |
| 12 | 1378 | 242 | 0.029 |  | 1539 | 271 | -0.044 |  | 1414 | 251 | 0.007 |
| 14 | 1849 | 347 | 0.069 |  | 2039 | 398 | 0.211 |  | 1846 | 338 | 0.201 |
| 16 | 2363 | 410 | 0.273 |  | 2539 | 477 | 0.300 |  | 2267 | 395 | 0.293 |
| 18 | 2669 | 421 | 0.079 |  | 2879 | 492 | -0.053 |  | 2446 | 395 | -0.100 |
| 20 | 2705 | 431 | -0.284 |  | 2951 | 498 | -0.299 |  | 2455 | 385 | -0.360 |
| **Females** | | | | | | | | | | | |
|  | White | | |  | Black | | |  | Mexican American | | |
| Age | M | σ | L |  | M | σ | L |  | M | σ | L |
| 8 | 888 | 132 | 0.030 |  | 925 | 145 | 0.677 |  | 833 | 112 | -0.157 |
| 10 | 1125 | 182 | 0.172 |  | 1271 | 210 | 0.495 |  | 1110 | 162 | -0.137 |
| 12 | 1445 | 243 | 0.321 |  | 1678 | 283 | 0.314 |  | 1471 | 229 | -0.116 |
| 14 | 1762 | 291 | 0.470 |  | 2016 | 329 | 0.132 |  | 1755 | 276 | -0.096 |
| 16 | 1971 | 309 | 0.619 |  | 2187 | 339 | -0.049 |  | 1899 | 291 | -0.076 |
| 18 | 2103 | 297 | 0.446 |  | 2285 | 328 | -0.219 |  | 1985 | 289 | -0.058 |
| 20 | 2124 | 286 | 0.138 |  | 2322 | 326 | -0.179 |  | 2011 | 282 | -0.017 |

M = Median, σ = Standard Deviation, L = Skewness (see LMS description in Methods).
