# Supplementary material for: Dual Energy X-Ray Absorptiometry Body Composition Reference Values from NHANES
Source: PLoS One. 2009 Sep 15;4(9):e7038. doi: 10.1371/journal.pone.0007038 (PMC2737140; doi:10.1371/journal.pone.0007038)
Supplement: Table S13 — Sub-total Body BMD (g/cm2) vs. Age in pediatric subjects. (0.05 MB DOC) [file pone.0007038.s033.doc]

Table S13: *Sub-total Body BMD (g/cm^2^) vs. Age in pediatric subjects.

| **Males** | | | | | | | | | | | | |
| --- | --- | --- | --- | --- | --- | --- | --- | --- | --- | --- | --- | --- |
|  | White | | | |  | Black | | |  | Mexican American | | |
| Age | M | | σ | L |  | M | σ | L |  | M | σ | L |
| 8 | 0.658 | | 0.054 | 2.080 |  | 0.680 | 0.061 | 0.846 |  | 0.636 | 0.059 | 0.426 |
| 10 | 0.714 | | 0.062 | 1.089 |  | 0.756 | 0.069 | 0.773 |  | 0.713 | 0.068 | 0.450 |
| 12 | 0.786 | | 0.073 | 0.360 |  | 0.840 | 0.080 | 0.590 |  | 0.799 | 0.077 | 0.498 |
| 14 | 0.898 | | 0.087 | 0.157 |  | 0.953 | 0.096 | 0.436 |  | 0.904 | 0.087 | 0.475 |
| 16 | 1.011 | | 0.097 | 0.364 |  | 1.067 | 0.109 | 0.486 |  | 1.000 | 0.095 | 0.332 |
| 18 | 1.076 | | 0.099 | 0.811 |  | 1.137 | 0.115 | 0.646 |  | 1.041 | 0.098 | 0.100 |
| 20 | 1.105 | | 0.096 | 1.392 |  | 1.180 | 0.117 | 0.763 |  | 1.042 | 0.097 | -0.176 |
| **Females** | | | | | | | | | | | | |
|  | | White | | |  | Black | | |  | Mexican American | | |
| Age | | M | σ | L |  | M | σ | L |  | M | σ | L |
| 8 | | 0.629 | 0.060 | 0.113 |  | 0.671 | 0.063 | 2.810 |  | 0.597 | 0.056 | -0.378 |
| 10 | | 0.724 | 0.068 | 0.270 |  | 0.781 | 0.071 | 1.910 |  | 0.715 | 0.065 | 0.304 |
| 12 | | 0.822 | 0.075 | 0.392 |  | 0.891 | 0.079 | 0.969 |  | 0.832 | 0.074 | 0.635 |
| 14 | | 0.900 | 0.078 | 0.408 |  | 0.969 | 0.082 | 0.192 |  | 0.906 | 0.077 | 0.592 |
| 16 | | 0.946 | 0.079 | 0.257 |  | 1.000 | 0.083 | -0.227 |  | 0.935 | 0.076 | 0.366 |
| 18 | | 0.965 | 0.079 | 0.136 |  | 1.011 | 0.084 | -0.404 |  | 0.939 | 0.075 | 0.055 |
| 20 | | 0.973 | 0.078 | 0.264 |  | 1.010 | 0.084 | -0.504 |  | 0.926 | 0.072 | -0.248 |

M = Median, σ = Standard Deviation, L = Skewness (see LMS description in Methods).

*Sub-total excludes head results.
