# Supplementary material for: Dual Energy X-Ray Absorptiometry Body Composition Reference Values from NHANES
Source: PLoS One. 2009 Sep 15;4(9):e7038. doi: 10.1371/journal.pone.0007038 (PMC2737140; doi:10.1371/journal.pone.0007038)
Supplement: Table S14 — Sub-total Body BMC (g) vs. Age in pediatric subjects. (0.05 MB DOC) [file pone.0007038.s034.doc]

Table S14: Sub-total Body BMC (g) vs. Age in pediatric subjects.

| **Males** | | | | | | | | | | | | | |
| --- | --- | --- | --- | --- | --- | --- | --- | --- | --- | --- | --- | --- | --- |
|  | White | | |  | Black | | |  | | Mexican American | | | |
| Age | M | σ | L |  | M | σ | L |  | | M | σ | L | |
| 8 | 651 | 130 | 0.030 |  | 708 | 133 | -0.334 |  | | 639 | 122 | -0.050 | |
| 10 | 803 | 159 | 0.096 |  | 899 | 173 | -0.172 |  | | 822 | 158 | 0.029 | |
| 12 | 1055 | 205 | 0.163 |  | 1193 | 235 | -0.011 |  | | 1099 | 212 | 0.110 | |
| 14 | 1493 | 286 | 0.230 |  | 1646 | 332 | 0.146 |  | | 1489 | 288 | 0.190 | |
| 16 | 1929 | 363 | 0.297 |  | 2078 | 426 | 0.297 |  | | 1836 | 354 | 0.265 | |
| 18 | 2172 | 402 | 0.363 |  | 2345 | 488 | 0.443 |  | | 1994 | 384 | 0.336 | |
| 20 | 2298 | 419 | 0.427 |  | 2520 | 531 | 0.580 |  | | 2024 | 388 | 0.403 | |
| **Females** | | | | | | | | | | | | | |
|  | White | | |  | Black | | | |  | Mexican American | | | |
| Age | M | σ | L |  | M | σ | L | |  | M | σ | | L |
| 8 | 623 | 127 | -0.031 |  | 656 | 134 | 0.696 | |  | 569 | 100 | | -0.044 |
| 10 | 841 | 166 | 0.107 |  | 967 | 190 | 0.502 | |  | 829 | 144 | | -0.067 |
| 12 | 1128 | 213 | 0.249 |  | 1323 | 250 | 0.309 | |  | 1147 | 198 | | -0.094 |
| 14 | 1393 | 252 | 0.386 |  | 1592 | 289 | 0.116 | |  | 1377 | 235 | | -0.124 |
| 16 | 1558 | 268 | 0.520 |  | 1715 | 298 | -0.074 | |  | 1473 | 248 | | -0.158 |
| 18 | 1648 | 270 | 0.653 |  | 1760 | 293 | -0.265 | |  | 1515 | 252 | | -0.194 |
| 20 | 1714 | 267 | 0.780 |  | 1764 | 281 | -0.448 | |  | 1511 | 248 | | -0.227 |

M = Median, σ = Standard Deviation, L = Skewness (see LMS description in Methods).

*Sub-total excludes head results.
