# Supplementary material for: Dual Energy X-Ray Absorptiometry Body Composition Reference Values from NHANES
Source: PLoS One. 2009 Sep 15;4(9):e7038. doi: 10.1371/journal.pone.0007038 (PMC2737140; doi:10.1371/journal.pone.0007038)
Supplement: Table S15 — Total Body BMD (g/cm2) vs. Height (cm) in pediatric subjects. (0.08 MB DOC) [file pone.0007038.s035.doc]

Table S15: Total Body BMD (g/cm^2^) vs. Height (cm) in pediatric subjects.

| **Males** | | | | | | | | | | | |
| --- | --- | --- | --- | --- | --- | --- | --- | --- | --- | --- | --- |
|  | White | | |  | Black | | |  | Mexican American | | |
| Height  (cm) | M | σ | L |  | M | σ | L |  | M | σ | L |
| 120 | 0.755 | 0.066 | 1.059 |  | - | - | - |  | - | - | - |
| 125 | 0.768 | 0.061 | 0.755 |  | 0.763 | 0.052 | 0.344 |  | 0.747 | 0.048 | 0.940 |
| 130 | 0.789 | 0.056 | 0.440 |  | 0.808 | 0.057 | 0.081 |  | 0.768 | 0.050 | 0.606 |
| 135 | 0.809 | 0.052 | 0.143 |  | 0.846 | 0.060 | -0.180 |  | 0.798 | 0.053 | 0.279 |
| 140 | 0.829 | 0.049 | -0.137 |  | 0.877 | 0.062 | -0.447 |  | 0.826 | 0.056 | -0.018 |
| 145 | 0.853 | 0.050 | -0.401 |  | 0.896 | 0.064 | -0.719 |  | 0.855 | 0.059 | -0.247 |
| 150 | 0.877 | 0.055 | -0.621 |  | 0.917 | 0.068 | -0.952 |  | 0.885 | 0.065 | -0.376 |
| 155 | 0.907 | 0.066 | -0.759 |  | 0.952 | 0.077 | -1.041 |  | 0.926 | 0.075 | -0.389 |
| 160 | 0.952 | 0.080 | -0.770 |  | 1.001 | 0.091 | -0.901 |  | 0.979 | 0.087 | -0.289 |
| 165 | 1.014 | 0.093 | -0.628 |  | 1.057 | 0.105 | -0.544 |  | 1.033 | 0.097 | -0.092 |
| 170 | 1.075 | 0.102 | -0.339 |  | 1.118 | 0.115 | -0.063 |  | 1.079 | 0.103 | 0.170 |
| 175 | 1.117 | 0.106 | 0.059 |  | 1.170 | 0.121 | 0.444 |  | 1.116 | 0.104 | 0.479 |
| 180 | 1.149 | 0.107 | 0.505 |  | 1.208 | 0.122 | 0.927 |  | 1.153 | 0.103 | 0.813 |
| 185 | 1.178 | 0.108 | 0.942 |  | 1.247 | 0.122 | 1.387 |  | 1.200 | 0.102 | 1.142 |
| 190 | 1.204 | 0.111 | 1.352 |  | 1.290 | 0.122 | 1.836 |  | 1.256 | 0.102 | 1.472 |
| 195 | 1.228 | 0.115 | 1.753 |  | 1.330 | 0.120 | 2.282 |  | - | - | - |
| 200 | - | - | - |  | 1.368 | 0.117 | 2.726 |  | - | - | - |
| **Females** | | | | | | | | | | | |
|  | White | | |  | Black | | |  | Mexican American | | |
| Height  (cm) | M | σ | L |  | M | σ | L |  | M | σ | L |
| 120 | - | - | - |  | - | - | - |  | 0.697 | 0.044 | -2.999 |
| 125 | 0.724 | 0.044 | -0.495 |  | 0.765 | 0.052 | -0.494 |  | 0.721 | 0.047 | -2.557 |
| 130 | 0.753 | 0.049 | -0.222 |  | 0.798 | 0.058 | -0.368 |  | 0.751 | 0.050 | -2.115 |
| 135 | 0.782 | 0.055 | 0.051 |  | 0.825 | 0.064 | -0.241 |  | 0.785 | 0.056 | -1.673 |
| 140 | 0.811 | 0.062 | 0.324 |  | 0.860 | 0.072 | -0.114 |  | 0.829 | 0.065 | -1.232 |
| 145 | 0.849 | 0.072 | 0.598 |  | 0.911 | 0.084 | 0.015 |  | 0.886 | 0.077 | -0.791 |
| 150 | 0.902 | 0.083 | 0.871 |  | 0.974 | 0.098 | 0.149 |  | 0.948 | 0.087 | -0.351 |
| 155 | 0.961 | 0.091 | 1.144 |  | 1.035 | 0.108 | 0.286 |  | 1.005 | 0.092 | 0.084 |
| 160 | 1.016 | 0.094 | 1.417 |  | 1.083 | 0.110 | 0.425 |  | 1.043 | 0.091 | 0.515 |
| 165 | 1.060 | 0.092 | 1.690 |  | 1.120 | 0.107 | 0.563 |  | 1.071 | 0.087 | 0.941 |
| 170 | 1.093 | 0.088 | 1.963 |  | 1.146 | 0.101 | 0.700 |  | 1.097 | 0.082 | 1.368 |
| 175 | 1.120 | 0.082 | 2.236 |  | 1.170 | 0.092 | 0.837 |  | 1.126 | 0.076 | 1.795 |
| 180 | 1.151 | 0.076 | 2.509 |  | 1.194 | 0.084 | 0.957 |  | 1.158 | 0.069 | 2.223 |
| 185 | - | - | - |  | - | - | - |  | 1.192 | 0.062 | 2.650 |

M = Median, σ = Standard Deviation, L = Skewness (see LMS description in Methods).
