# Supplementary material for: Dual Energy X-Ray Absorptiometry Body Composition Reference Values from NHANES
Source: PLoS One. 2009 Sep 15;4(9):e7038. doi: 10.1371/journal.pone.0007038 (PMC2737140; doi:10.1371/journal.pone.0007038)
Supplement: Table S16 — Total Body BMC (g) vs. Height (cm) in pediatric subjects. (0.09 MB DOC) [file pone.0007038.s036.doc]

Table S16: Total Body BMC (g) vs. Height (cm) in pediatric subjects.

| **Males** | | | | | | | | | | | |
| --- | --- | --- | --- | --- | --- | --- | --- | --- | --- | --- | --- |
|  | White | | |  | Black | | |  | Mexican American | | |
| Height  (cm) | M | σ | L |  | M | σ | L |  | M | σ | L |
| 120 | 832 | 87 | 1.803 |  | - | - | - |  | - | - | - |
| 125 | 899 | 89 | 1.458 |  | 906 | 56 | -0.198 |  | 897 | 81 | 1.894 |
| 130 | 975 | 92 | 1.101 |  | 1014 | 78 | -0.265 |  | 971 | 84 | 1.620 |
| 135 | 1039 | 95 | 0.754 |  | 1112 | 97 | -0.335 |  | 1045 | 89 | 1.354 |
| 140 | 1118 | 102 | 0.425 |  | 1211 | 115 | -0.418 |  | 1133 | 101 | 1.107 |
| 145 | 1213 | 115 | 0.130 |  | 1303 | 134 | -0.532 |  | 1243 | 121 | 0.901 |
| 150 | 1326 | 138 | -0.116 |  | 1424 | 162 | -0.658 |  | 1381 | 154 | 0.753 |
| 155 | 1479 | 176 | -0.308 |  | 1592 | 204 | -0.732 |  | 1569 | 199 | 0.661 |
| 160 | 1681 | 224 | -0.427 |  | 1810 | 257 | -0.700 |  | 1794 | 249 | 0.613 |
| 165 | 1934 | 273 | -0.447 |  | 2058 | 313 | -0.563 |  | 2025 | 293 | 0.595 |
| 170 | 2200 | 313 | -0.365 |  | 2328 | 360 | -0.356 |  | 2248 | 325 | 0.582 |
| 175 | 2435 | 338 | -0.210 |  | 2592 | 392 | -0.107 |  | 2459 | 344 | 0.578 |
| 180 | 2643 | 357 | -0.030 |  | 2840 | 414 | 0.155 |  | 2678 | 359 | 0.577 |
| 185 | 2837 | 373 | 0.138 |  | 3096 | 435 | 0.418 |  | 2912 | 376 | 0.562 |
| 190 | 3022 | 389 | 0.287 |  | 3358 | 452 | 0.680 |  | 3158 | 395 | 0.539 |
| 195 | 3204 | 404 | 0.428 |  | 3618 | 464 | 0.941 |  | - | - | - |
| 200 | - | - | - |  | 3877 | 471 | 1.200 |  | - | - | - |
| **Females** | | | | | | | | | | | |
|  | White | | |  | Black | | |  | Mexican American | | |
| Height  (cm) | M | σ | L |  | M | σ | L |  | M | σ | L |
| 120 | - | - | - |  | - | - | - |  | 786 | 47 | 0.003 |
| 125 | 845 | 63 | 0.811 |  | 898 | 108 | -0.019 |  | 855 | 69 | 0.003 |
| 130 | 904 | 81 | 0.811 |  | 980 | 118 | -0.019 |  | 933 | 90 | 0.003 |
| 135 | 985 | 102 | 0.811 |  | 1069 | 133 | -0.019 |  | 1021 | 109 | 0.003 |
| 140 | 1080 | 127 | 0.811 |  | 1187 | 161 | -0.019 |  | 1139 | 137 | 0.003 |
| 145 | 1214 | 163 | 0.811 |  | 1354 | 202 | -0.019 |  | 1307 | 177 | 0.003 |
| 150 | 1395 | 207 | 0.811 |  | 1568 | 251 | -0.019 |  | 1512 | 216 | 0.003 |
| 155 | 1607 | 246 | 0.811 |  | 1794 | 295 | -0.019 |  | 1716 | 239 | 0.003 |
| 160 | 1820 | 264 | 0.811 |  | 2005 | 317 | -0.019 |  | 1893 | 246 | 0.003 |
| 165 | 2019 | 269 | 0.811 |  | 2209 | 329 | -0.019 |  | 2066 | 253 | 0.003 |
| 170 | 2198 | 274 | 0.811 |  | 2404 | 331 | -0.019 |  | 2246 | 266 | 0.003 |
| 175 | 2371 | 272 | 0.811 |  | 2604 | 322 | -0.019 |  | 2437 | 285 | 0.003 |
| 180 | 2553 | 262 | 0.811 |  | 2782 | 311 | -0.019 |  | 2638 | 313 | 0.003 |
| 185 | - | - | - |  | - | - | - |  | 2846 | 349 | 0.003 |

M = Median, σ = Standard Deviation, L = Skewness (see LMS description in Methods).
