# Supplementary material for: Dual Energy X-Ray Absorptiometry Body Composition Reference Values from NHANES
Source: PLoS One. 2009 Sep 15;4(9):e7038. doi: 10.1371/journal.pone.0007038 (PMC2737140; doi:10.1371/journal.pone.0007038)
Supplement: Table S17 — Sub-total Body BMD (g/cm2) vs. Height (cm) in pediatric subjects. (0.08 MB DOC) [file pone.0007038.s037.doc]

Table S17: Sub-total Body BMD (g/cm^2^) vs. Height (cm) in pediatric subjects.

| **Males** | | | | | | | | | | | |
| --- | --- | --- | --- | --- | --- | --- | --- | --- | --- | --- | --- |
|  | White | | |  | Black | | |  | Mexican American | | |
| Height  (cm) | M | σ | L |  | M | σ | L |  | M | σ | L |
| 125 | 0.634 | 0.047 | 1.728 |  | 0.623 | 0.044 | 0.484 |  | 0.616 | 0.041 | 0.842 |
| 130 | 0.662 | 0.046 | 1.345 |  | 0.670 | 0.048 | 0.232 |  | 0.646 | 0.045 | 0.691 |
| 135 | 0.688 | 0.045 | 0.974 |  | 0.713 | 0.051 | -0.022 |  | 0.682 | 0.048 | 0.535 |
| 140 | 0.713 | 0.046 | 0.613 |  | 0.751 | 0.055 | -0.279 |  | 0.716 | 0.052 | 0.386 |
| 145 | 0.741 | 0.048 | 0.270 |  | 0.780 | 0.059 | -0.539 |  | 0.752 | 0.056 | 0.262 |
| 150 | 0.774 | 0.053 | -0.027 |  | 0.812 | 0.065 | -0.756 |  | 0.794 | 0.062 | 0.182 |
| 155 | 0.814 | 0.061 | -0.253 |  | 0.856 | 0.074 | -0.837 |  | 0.844 | 0.070 | 0.157 |
| 160 | 0.866 | 0.072 | -0.369 |  | 0.910 | 0.085 | -0.713 |  | 0.899 | 0.079 | 0.193 |
| 165 | 0.927 | 0.082 | -0.340 |  | 0.970 | 0.097 | -0.396 |  | 0.951 | 0.087 | 0.296 |
| 170 | 0.986 | 0.091 | -0.165 |  | 1.029 | 0.107 | 0.034 |  | 0.994 | 0.093 | 0.443 |
| 175 | 1.030 | 0.097 | 0.118 |  | 1.081 | 0.114 | 0.491 |  | 1.032 | 0.096 | 0.622 |
| 180 | 1.064 | 0.101 | 0.446 |  | 1.122 | 0.118 | 0.925 |  | 1.071 | 0.098 | 0.816 |
| 185 | 1.094 | 0.105 | 0.768 |  | 1.163 | 0.122 | 1.333 |  | 1.115 | 0.101 | 1.003 |
| 190 | 1.121 | 0.108 | 1.064 |  | 1.206 | 0.125 | 1.726 |  | 1.163 | 0.103 | 1.190 |
| 195 | 1.146 | 0.112 | 1.348 |  | 1.247 | 0.128 | 2.115 |  | - | - | - |
| 200 | 1.170 | 0.115 | 1.635 |  | 1.287 | 0.130 | 2.502 |  | - | - | - |
| **Females** | | | | | | | | | | | |
|  | White | | |  | Black | | |  | Mexican American | | |
| Height  (cm) | M | σ | L |  | M | σ | L |  | M | σ | L |
| 125 | 0.606 | 0.035 | -1.623 |  | 0.648 | 0.053 | 0.713 |  | 0.605 | 0.040 | -2.980 |
| 130 | 0.639 | 0.042 | -0.986 |  | 0.686 | 0.056 | 0.699 |  | 0.639 | 0.044 | -2.237 |
| 135 | 0.676 | 0.049 | -0.337 |  | 0.716 | 0.059 | 0.649 |  | 0.679 | 0.050 | -1.549 |
| 140 | 0.708 | 0.056 | 0.336 |  | 0.755 | 0.066 | 0.586 |  | 0.725 | 0.056 | -0.892 |
| 145 | 0.746 | 0.065 | 0.980 |  | 0.807 | 0.076 | 0.557 |  | 0.781 | 0.064 | -0.226 |
| 150 | 0.795 | 0.076 | 1.492 |  | 0.865 | 0.086 | 0.603 |  | 0.841 | 0.070 | 0.394 |
| 155 | 0.851 | 0.084 | 1.807 |  | 0.918 | 0.093 | 0.695 |  | 0.894 | 0.074 | 0.807 |
| 160 | 0.905 | 0.086 | 1.882 |  | 0.959 | 0.095 | 0.737 |  | 0.928 | 0.074 | 0.952 |
| 165 | 0.949 | 0.084 | 1.829 |  | 0.993 | 0.094 | 0.701 |  | 0.955 | 0.074 | 0.910 |
| 170 | 0.981 | 0.081 | 1.684 |  | 1.021 | 0.091 | 0.612 |  | 0.981 | 0.074 | 0.867 |
| 175 | 1.010 | 0.077 | 1.493 |  | 1.053 | 0.088 | 0.467 |  | 1.011 | 0.073 | 0.860 |
| 180 | 1.045 | 0.073 | 1.290 |  | 1.084 | 0.084 | 0.325 |  | 1.045 | 0.072 | 0.864 |
| 185 | - | - | - |  | - | - | - |  | 1.083 | 0.072 | 0.873 |

M = Median, σ = Standard Deviation, L = Skewness (see LMS description in Methods).

*Sub-total excludes head results.
