# Supplementary material for: Dual Energy X-Ray Absorptiometry Body Composition Reference Values from NHANES
Source: PLoS One. 2009 Sep 15;4(9):e7038. doi: 10.1371/journal.pone.0007038 (PMC2737140; doi:10.1371/journal.pone.0007038)
Supplement: Table S18 — Sub-total BMC (g) vs. Height (cm) in pediatric subjects. (0.08 MB DOC) [file pone.0007038.s038.doc]

Table S18: Sub-total BMC (g) vs. Height (cm) in pediatric subjects.

| **Males** | | | | | | | | | | | | | |
| --- | --- | --- | --- | --- | --- | --- | --- | --- | --- | --- | --- | --- | --- |
|  | White | | |  | Black | | | |  | | Mexican American | | |
| Height  (cm) | M | σ | L |  | M | σ | L | |  | | M | σ | L |
| 125 | 606 | 67 | 1.154 |  | 605 | 48 | -0.788 | |  | | 609 | 61 | 1.633 |
| 130 | 673 | 72 | 0.921 |  | 692 | 61 | -0.733 | |  | | 673 | 67 | 1.379 |
| 135 | 733 | 77 | 0.691 |  | 779 | 76 | -0.676 | |  | | 744 | 75 | 1.147 |
| 140 | 803 | 85 | 0.463 |  | 869 | 93 | -0.623 | |  | | 823 | 87 | 0.951 |
| 145 | 888 | 98 | 0.245 |  | 959 | 112 | -0.604 | |  | | 924 | 105 | 0.816 |
| 150 | 997 | 119 | 0.049 |  | 1079 | 139 | -0.612 | |  | | 1061 | 131 | 0.743 |
| 155 | 1145 | 149 | -0.117 |  | 1239 | 177 | -0.599 | |  | | 1238 | 167 | 0.715 |
| 160 | 1333 | 186 | -0.235 |  | 1439 | 222 | -0.529 | |  | | 1439 | 207 | 0.715 |
| 165 | 1555 | 226 | -0.276 |  | 1665 | 270 | -0.398 | |  | | 1642 | 244 | 0.724 |
| 170 | 1787 | 263 | -0.223 |  | 1904 | 313 | -0.214 | |  | | 1838 | 275 | 0.719 |
| 175 | 1999 | 292 | -0.101 |  | 2140 | 348 | 0.015 | |  | | 2030 | 299 | 0.707 |
| 180 | 2192 | 313 | 0.050 |  | 2369 | 375 | 0.263 | |  | | 2227 | 320 | 0.692 |
| 185 | 2372 | 331 | 0.201 |  | 2603 | 400 | 0.514 | |  | | 2432 | 340 | 0.661 |
| 190 | 2547 | 346 | 0.340 |  | 2839 | 421 | 0.762 | |  | | 2642 | 359 | 0.622 |
| 195 | 2719 | 360 | 0.473 |  | 3075 | 440 | 1.008 | |  | | - | - | - |
| 200 | 2890 | 371 | 0.606 |  | 3310 | 455 | 1.253 | |  | | - | - | - |
| **Females** | | | | | | | | | | | | | |
|  | White | | |  | Black | | | | |  | Mexican American | | |
| Height  (cm) | M | σ | L |  | M | σ | | L | |  | M | σ | L |
| 120 | - | - | - |  | - | - | | - | |  | 527 | 43 | 0.370 |
| 125 | 587 | 44 | 1.191 |  | 631 | 87 | | 0.560 | |  | 593 | 56 | 0.147 |
| 130 | 640 | 61 | 1.034 |  | 699 | 97 | | 0.301 | |  | 664 | 71 | -0.068 |
| 135 | 715 | 82 | 0.897 |  | 776 | 111 | | 0.077 | |  | 742 | 88 | -0.236 |
| 140 | 797 | 104 | 0.834 |  | 883 | 134 | | -0.060 | |  | 841 | 108 | -0.299 |
| 145 | 908 | 132 | 0.841 |  | 1027 | 164 | | -0.060 | |  | 980 | 133 | -0.215 |
| 150 | 1061 | 165 | 0.882 |  | 1208 | 200 | | 0.041 | |  | 1157 | 161 | -0.018 |
| 155 | 1242 | 197 | 0.930 |  | 1393 | 231 | | 0.130 | |  | 1333 | 182 | 0.198 |
| 160 | 1427 | 219 | 0.942 |  | 1565 | 253 | | 0.084 | |  | 1481 | 195 | 0.276 |
| 165 | 1601 | 228 | 0.911 |  | 1733 | 269 | | -0.067 | |  | 1632 | 210 | 0.178 |
| 170 | 1757 | 232 | 0.853 |  | 1903 | 281 | | -0.257 | |  | 1791 | 227 | 0.024 |
| 175 | 1909 | 231 | 0.798 |  | 2092 | 290 | | -0.469 | |  | 1965 | 248 | -0.134 |
| 180 | 2076 | 228 | 0.745 |  | 2261 | 295 | | -0.660 | |  | 2152 | 272 | -0.301 |
| 185 | - | - | - |  | - | - | | - | |  | 2349 | 300 | -0.475 |

M = Median, σ = Standard Deviation, L = Skewness (see LMS description in Methods).

*Sub-total excludes head results.
