# Supplementary material for: Dual Energy X-Ray Absorptiometry Body Composition Reference Values from NHANES
Source: PLoS One. 2009 Sep 15;4(9):e7038. doi: 10.1371/journal.pone.0007038 (PMC2737140; doi:10.1371/journal.pone.0007038)
Supplement: Table S19 — Total Lean Mass (g) vs. Height (cm) in pediatric subjects. (0.08 MB DOC) [file pone.0007038.s039.doc]

Table S19: Total Lean Mass (g) vs. Height (cm) in pediatric subjects.

| **Males** | | | | | | | | | | | |
| --- | --- | --- | --- | --- | --- | --- | --- | --- | --- | --- | --- |
|  | White | | |  | Black | | |  | Mexican American | | |
| Height  (cm) | M | σ | L |  | M | σ | L |  | M | σ | L |
| 120 | 17311 | 1703 | -0.187 |  | - | - | - |  | - | - | - |
| 125 | 19267 | 1908 | -0.263 |  | 19261 | 1575 | -1.429 |  | 19300 | 1862 | -1.117 |
| 130 | 21577 | 2154 | -0.341 |  | 21732 | 1934 | -1.380 |  | 21404 | 2222 | -1.015 |
| 135 | 23912 | 2418 | -0.419 |  | 24230 | 2325 | -1.331 |  | 23703 | 2619 | -0.913 |
| 140 | 26065 | 2691 | -0.498 |  | 26820 | 2753 | -1.282 |  | 26316 | 3060 | -0.812 |
| 145 | 28362 | 3016 | -0.576 |  | 29422 | 3228 | -1.233 |  | 29356 | 3564 | -0.710 |
| 150 | 31130 | 3434 | -0.655 |  | 32917 | 3864 | -1.184 |  | 33059 | 4172 | -0.608 |
| 155 | 34821 | 3989 | -0.733 |  | 37057 | 4640 | -1.135 |  | 37415 | 4895 | -0.506 |
| 160 | 39479 | 4682 | -0.811 |  | 41523 | 5497 | -1.086 |  | 42196 | 5700 | -0.404 |
| 165 | 44796 | 5473 | -0.890 |  | 46335 | 6403 | -1.037 |  | 47001 | 6515 | -0.302 |
| 170 | 50113 | 6279 | -0.968 |  | 51432 | 7323 | -0.987 |  | 51472 | 7281 | -0.200 |
| 175 | 54835 | 7022 | -1.047 |  | 56349 | 8183 | -0.938 |  | 55735 | 8015 | -0.098 |
| 180 | 59000 | 7700 | -1.125 |  | 60954 | 8984 | -0.889 |  | 60185 | 8779 | 0.004 |
| 185 | 62936 | 8351 | -1.203 |  | 65512 | 9781 | -0.840 |  | 64774 | 9579 | 0.106 |
| 190 | 66842 | 9002 | -1.282 |  | 70147 | 10598 | -0.791 |  | 69384 | 10403 | 0.208 |
| 195 | 70750 | 9663 | -1.360 |  | 74799 | 11432 | -0.742 |  | - | - | - |
| 200 | 74671 | 10336 | -1.439 |  | 79442 | 12280 | -0.693 |  | - | - | - |
| **Females** | | | | | | | | | | | |
|  | White | | |  | Black | | |  | Mexican American | | |
| Height  (cm) | M | σ | L |  | M | σ | L |  | M | σ | L |
| 120 | - | - | - |  | - | - | - |  | 15690 | 1442 | -1.540 |
| 125 | 17256 | 1759 | -1.206 |  | 18632 | 2221 | -1.381 |  | 17731 | 1766 | -1.540 |
| 130 | 19696 | 2109 | -1.206 |  | 21240 | 2664 | -1.381 |  | 19872 | 2129 | -1.540 |
| 135 | 22324 | 2504 | -1.206 |  | 23946 | 3158 | -1.381 |  | 22317 | 2537 | -1.540 |
| 140 | 25217 | 2952 | -1.206 |  | 26976 | 3714 | -1.381 |  | 25186 | 2979 | -1.540 |
| 145 | 28420 | 3440 | -1.206 |  | 30324 | 4297 | -1.381 |  | 28440 | 3437 | -1.540 |
| 150 | 31789 | 3921 | -1.206 |  | 33840 | 4867 | -1.381 |  | 31876 | 3912 | -1.540 |
| 155 | 35101 | 4339 | -1.206 |  | 37331 | 5408 | -1.381 |  | 35266 | 4435 | -1.540 |
| 160 | 38240 | 4673 | -1.206 |  | 40770 | 5940 | -1.381 |  | 38579 | 5035 | -1.540 |
| 165 | 41183 | 4950 | -1.206 |  | 44258 | 6538 | -1.381 |  | 41977 | 5717 | -1.540 |
| 170 | 43993 | 5203 | -1.206 |  | 47885 | 7221 | -1.381 |  | 45489 | 6456 | -1.540 |
| 175 | 46760 | 5441 | -1.206 |  | 51578 | 7960 | -1.381 |  | 49067 | 7245 | -1.540 |
| 180 | 49534 | 5670 | -1.206 |  | 54828 | 8642 | -1.381 |  | 52676 | 8085 | -1.540 |
| 185 | - | - | - |  | - | - | - |  | 56298 | 8974 | -1.540 |

M = Median, σ = Standard Deviation, L = Skewness (see LMS description in Methods).
