# Supplementary material for: Dual Energy X-Ray Absorptiometry Body Composition Reference Values from NHANES
Source: PLoS One. 2009 Sep 15;4(9):e7038. doi: 10.1371/journal.pone.0007038 (PMC2737140; doi:10.1371/journal.pone.0007038)
Supplement: Table S20 — sub-total BMC (g) vs. Total Lean Mass (g) in pediatric subjects. (0.09 MB DOC) [file pone.0007038.s040.doc]

Table S20: Sub-total BMC (g) vs. Total Lean Mass (g) in pediatric subjects.

| **Males** | | | | | | | | | | | | | |
| --- | --- | --- | --- | --- | --- | --- | --- | --- | --- | --- | --- | --- | --- |
|  | White | | |  | Black | | |  | Mexican American | | | | |
| Height  (cm) | M | σ | L |  | M | σ | L |  | M | | σ | | L |
| 15000 | 485 | 48 | 0.480 |  | - | - | - |  | - | | - | | - |
| 20000 | 634 | 63 | 0.480 |  | 655 | 62 | 0.148 |  | 647 | | 69 | | 0.847 |
| 25000 | 777 | 78 | 0.480 |  | 816 | 79 | 0.148 |  | 785 | | 84 | | 0.847 |
| 30000 | 942 | 94 | 0.480 |  | 974 | 97 | 0.148 |  | 939 | | 100 | | 0.847 |
| 35000 | 1133 | 114 | 0.480 |  | 1155 | 118 | 0.148 |  | 1129 | | 120 | | 0.847 |
| 40000 | 1352 | 137 | 0.480 |  | 1371 | 143 | 0.148 |  | 1347 | | 143 | | 0.847 |
| 45000 | 1587 | 161 | 0.480 |  | 1604 | 171 | 0.148 |  | 1571 | | 166 | | 0.847 |
| 50000 | 1820 | 185 | 0.480 |  | 1847 | 201 | 0.148 |  | 1786 | | 188 | | 0.847 |
| 55000 | 2027 | 207 | 0.480 |  | 2085 | 231 | 0.148 |  | 1973 | | 207 | | 0.847 |
| 60000 | 2204 | 227 | 0.480 |  | 2298 | 260 | 0.148 |  | 2136 | | 224 | | 0.847 |
| 65000 | 2357 | 243 | 0.480 |  | 2479 | 286 | 0.148 |  | 2288 | | 239 | | 0.847 |
| 70000 | 2493 | 258 | 0.480 |  | 2637 | 310 | 0.148 |  | 2427 | | 253 | | 0.847 |
| 75000 | 2622 | 273 | 0.480 |  | 2779 | 333 | 0.148 |  | 2558 | | 266 | | 0.847 |
| 80000 | 2748 | 287 | 0.480 |  | 2907 | 355 | 0.148 |  | 2687 | | 279 | | 0.847 |
| 85000 | 2871 | 301 | 0.480 |  | 3031 | 376 | 0.148 |  | 2816 | | 292 | | 0.847 |
| 90000 | 2995 | 316 | 0.480 |  | 3154 | 399 | 0.148 |  | 2946 | | 304 | | 0.847 |
| 95000 | - | - | - |  | 3275 | 421 | 0.148 |  | 3077 | | 317 | | 0.847 |
| 100000 | - | - | - |  | 3395 | 444 | 0.148 |  | 3210 | | 330 | | 0.847 |
| 105000 | - | - | - |  | 3514 | 467 | 0.148 |  | - | | - | | - |
| 110000 | - | - | - |  | 3633 | 491 | 0.148 |  | - | | - | | - |
| **Females** | | | | | | | | | | | | | |
|  | White | | |  | Black | | |  | Mexican American | | | | |
| Height  (cm) | M | σ | L |  | M | σ | L |  | M | σ | | L | |
| 15000 | 527 | 56 | 2.121 |  | 532 | 65 | 1.190 |  | 520 | 61 | | 1.495 | |
| 20000 | 657 | 73 | 2.121 |  | 676 | 82 | 1.190 |  | 678 | 78 | | 1.495 | |
| 25000 | 806 | 94 | 2.121 |  | 830 | 100 | 1.190 |  | 853 | 96 | | 1.495 | |
| 30000 | 1032 | 124 | 2.121 |  | 1035 | 124 | 1.190 |  | 1087 | 121 | | 1.495 | |
| 35000 | 1279 | 160 | 2.121 |  | 1300 | 155 | 1.190 |  | 1330 | 145 | | 1.495 | |
| 40000 | 1511 | 195 | 2.121 |  | 1544 | 183 | 1.190 |  | 1522 | 162 | | 1.495 | |
| 45000 | 1692 | 225 | 2.121 |  | 1731 | 203 | 1.190 |  | 1659 | 173 | | 1.495 | |
| 50000 | 1855 | 253 | 2.121 |  | 1877 | 218 | 1.190 |  | 1773 | 181 | | 1.495 | |
| 55000 | 1998 | 280 | 2.121 |  | 1999 | 231 | 1.190 |  | 1905 | 190 | | 1.495 | |
| 60000 | 2125 | 306 | 2.121 |  | 2127 | 243 | 1.190 |  | 2057 | 201 | | 1.495 | |
| 65000 | 2246 | 331 | 2.121 |  | 2271 | 257 | 1.190 |  | 2230 | 213 | | 1.495 | |
| 70000 | - | - | - |  | 2424 | 272 | 1.190 |  | 2421 | 226 | | 1.495 | |
| 75000 | - | - | - |  | 2577 | 287 | 1.190 |  | 2624 | 239 | | 1.495 | |
| 80000 | - | - | - |  | 2736 | 302 | 1.190 |  | - | - | | - | |
| 85000 | - | - | - |  | 2899 | 317 | 1.190 |  | - | - | | - | |

M = Median, σ = Standard Deviation, L = Skewness (see LMS description in Methods).

*Sub-total excludes head results.
